# Supplementary material for: Modeling liver cancer molecular and histological features by CRISPR editing of porcine hepatocytes
Source: JHEP Rep. 2026 Sep 23;8(12):102042. doi: 10.1016/j.jhepr.2026.102042 (PMC13770662; doi:10.1016/j.jhepr.2026.102042)
Supplement: Supplementary file 1 [file mmc1.pdf]

# Modeling liver cancer molecular and histological features by CRISPR editing of porcine hepatocytes

Lobna Elkhadragy, Olayinka G. David, Caitlyn C. Castillo, Luke N. Redlon, Luke R. Jordan, Nusrat Khan, Hannah Liu, Isadora Andre Rosa Lopes, Yanqiong Zhou, Hiroaki Kanzaki, Yujin Hoshida, Jonathan P. Samuelson, Amaia Lujambio, Lawrence B. Schook, Grace Guzman, Kyle M. Schachtschneider, Ron C. Gaba

Table of contents

Supplementary Materials and Methods……………………………………………………………2

Supplementary Figures…………………………………….……………………………………..4

Supplementary Tables…………………………………………...……...separate excel file

## Supplementary Materials and Methods

**Western blotting:** Cells were washed with ice-cold PBS and lysed with RIPA buffer (Thermo Fisher #AAJ63306AK) containing protease inhibitor cocktail (Thermo Fisher #78429). Protein lysates were resolved by SDS–PAGE on 4-20% gels, transferred to nitrocellulose membranes, and blocked with 5% non-fat milk in PBS with tween 20 for 30 minutes. Membranes were incubated with primary antibodies overnight at 4°C, followed by 1 hour incubation with HRP-conjugated secondary antibodies at room temperature. The following antibodies were used: anti-c-Myc (Santa Cruz #9E10, sc-40), anti-PTEN (Santa Cruz sc-7974), anti-β-actin (Santa Cruz sc-47778), and anti-mouse secondary antibody (Biorad #170-6516). The Western blots were visualized by chemiluminescence (Thermo Fisher #32109).

**Doubling time analysis:** In vitro doubling time was determined by counting live cells that did not uptake Trypan blue dye at two time points and using the equation: doubling time = $T \ln 2/\ln(X2/X1)$, where T is the incubation time, X1 is the initial cell number, and X2 is the final cell number. Results were expressed as mean ± standard deviation (SD). Statistical comparisons were performed by one-way analysis of variance (ANOVA) using GraphPad Prism 10, with $p < 0.05$ considered statistically significant.

**Isolation of single-cell clones:** Cells were diluted to 1 cell/100 µl medium and seeded into 96-well plates. Wells were inspected microscopically to confirm the presence of a single cell/colony per well. Colonies reaching ~80-90% confluence were split into two wells: one for genomic DNA extraction and indel analysis, and the other for expansion.

**Histology and immunohistochemistry (IHC)**: Formalin-fixed, paraffin-embedded tissues were sectioned and stained with hematoxylin and eosin (H&E) and subjected to

IHC using antibodies against Arginase (Abcam #ab91279), CK19 (DSHB, Troma-III), c-Myc (Biocare Medical #PME415AA), and Ki67 (Agilent # M7240, MIB-1 clone). Whole slides were scanned at 20x magnification using the Leica Aperio AT2 slide scanner and digital images were visualized with Aperio ImageScope software (Leica).

**Ki67 quantification**: Digitized slides were analyzed in Halo (Indica Labs, v4.0.5107.488). Tissue segmentation into epithelium and stroma was performed using an AI-based mininet classifier, with three classifier sets required for segmenting all tissue in the analysis. Ki67 positivity was quantified in the tumor compartment using the Multiplex IHC v3.4.9 algorithm, with thresholds manually set and applied consistently across all samples. Tissue microarrays containing one or two representative tumor cores per tumor were analyzed. Data is expressed as the percentage of Ki67-positive tumor cells in tumor sections.

**Supplementary Figures**

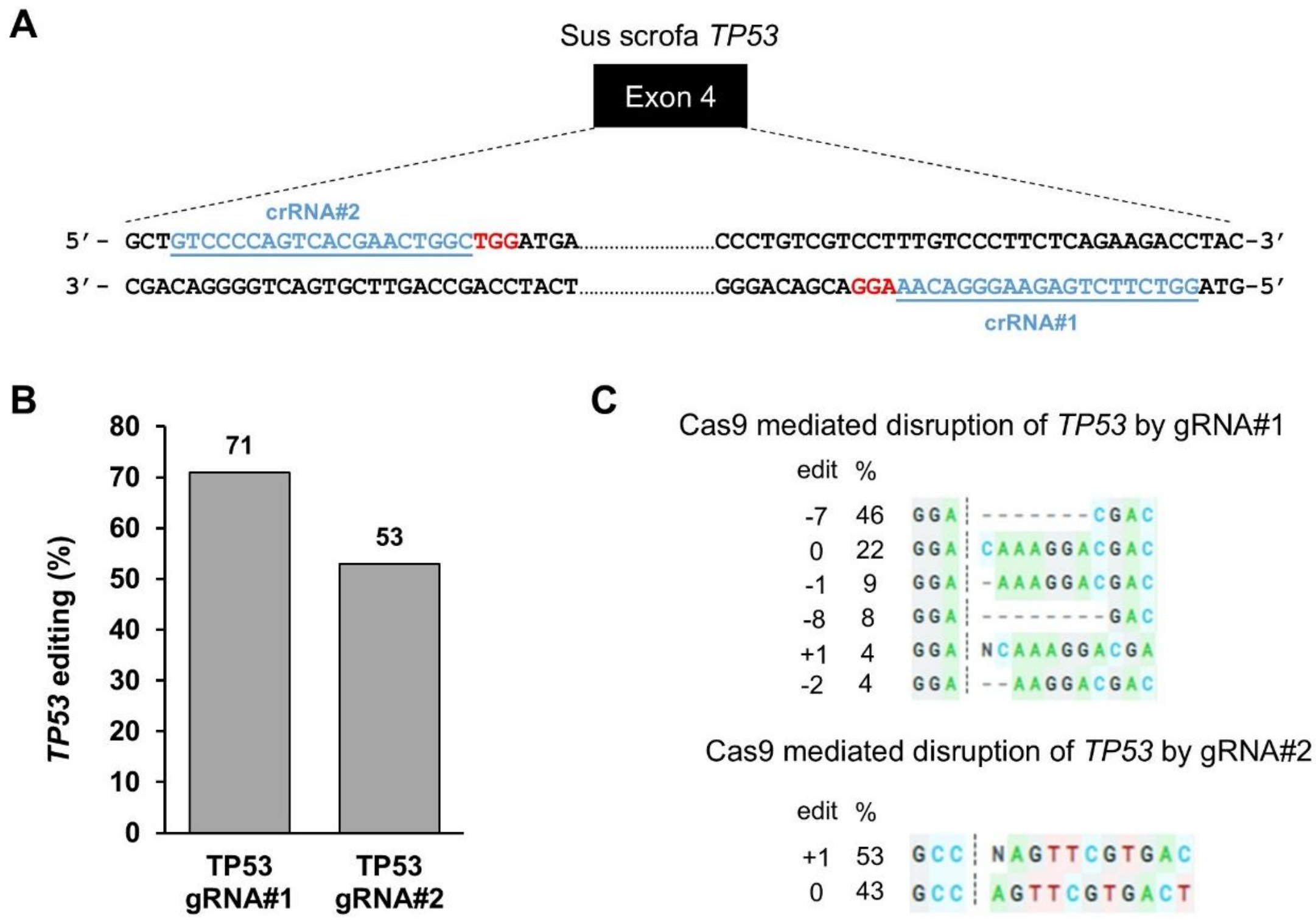


**Fig. S1. Screening CRISPR/Cas9 sgRNAs targeting porcine *TP53* gene.**

A) Schematic of the porcine *TP53* locus showing CRISPR spacer sequences crRNA#1 and crRNA#2 (blue, underlined). Protospacer adjacent motif (PAM) sequences are marked in red. B) Comparison of editing efficiency between the two *TP53*-targeting sgRNAs. Porcine cells (Oncopig A343 HCC cells, ref. #21) were transfected with each Cas9-sgRNA ribonucleoprotein complex and genomic DNA was extracted two days post-transfection for Sanger sequencing. Non-transfected cells were used as control. The bar graph shows the percentage (%) of reads with indels at each sgRNA target site, reflecting non-homologous end joining (NHEJ). C) Type and frequency of *TP53* indels detected by sequencing analysis. The percentage of reads for each sequence are shown on the left. Dashed line, predicted Cas9 cleavage position; N, nucleotide insertion; dash, deleted base.

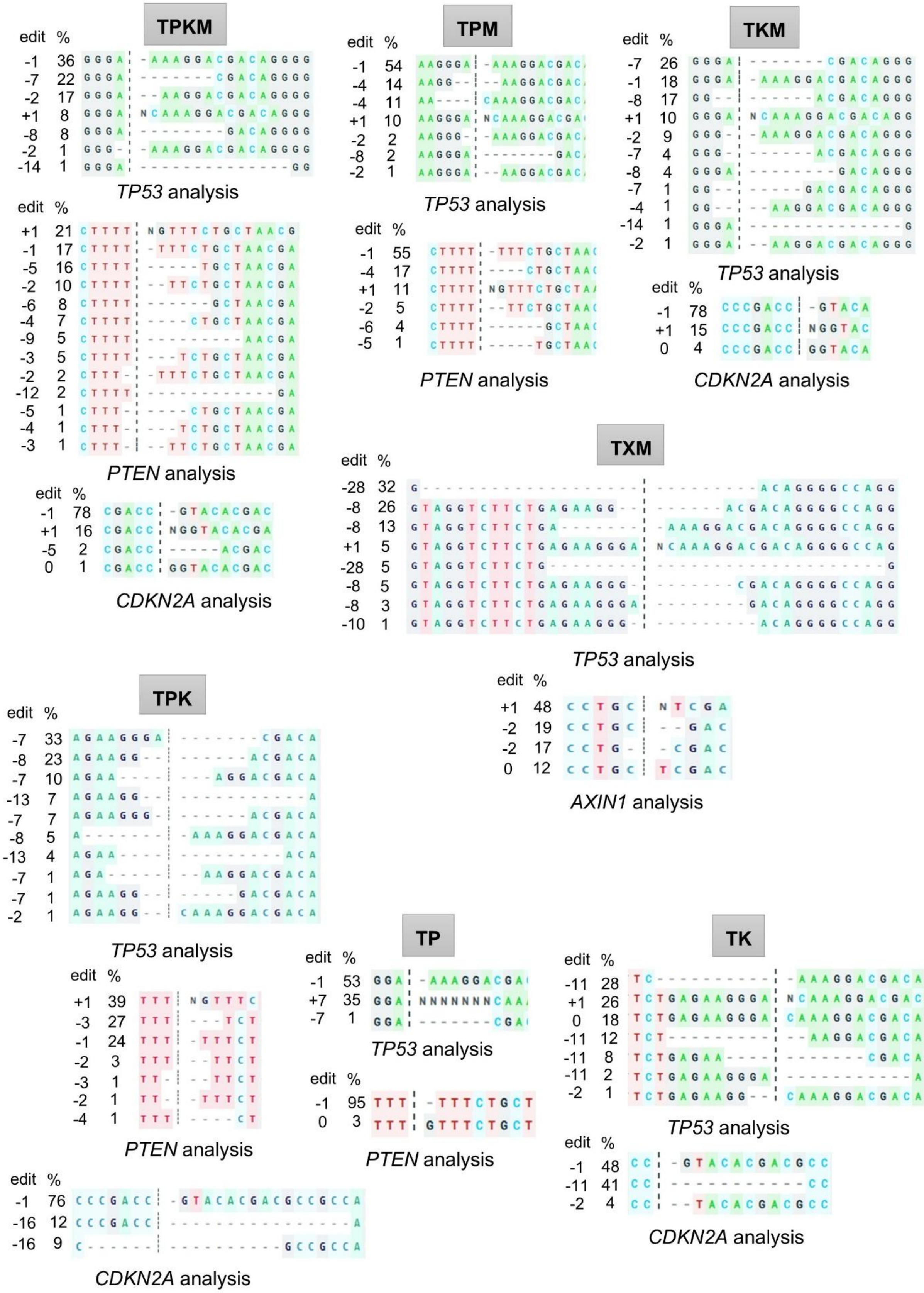


**Fig. S2. Validation of CRISPR gene editing in developed cell lines by sequencing analysis.**

Sequencing analysis confirmed CRISPR-induced edits in the targeted genes in the established cell lines. Representative indel analysis is shown for the following cells at the indicated passage (p) number, with pig of origin indicated: PG1-TPKM (p.8), PG1-TPM (p.9), PG1-TKM (p.5), PG2-TPK (p.17), PG1-TXM (p.17), PG3-TP (p.11), and PG3-TK (p.23). The type and frequency of indels are shown on the left. Dashed line, predicted Cas9 cleavage position; N, nucleotide insertion; dash, deleted base.

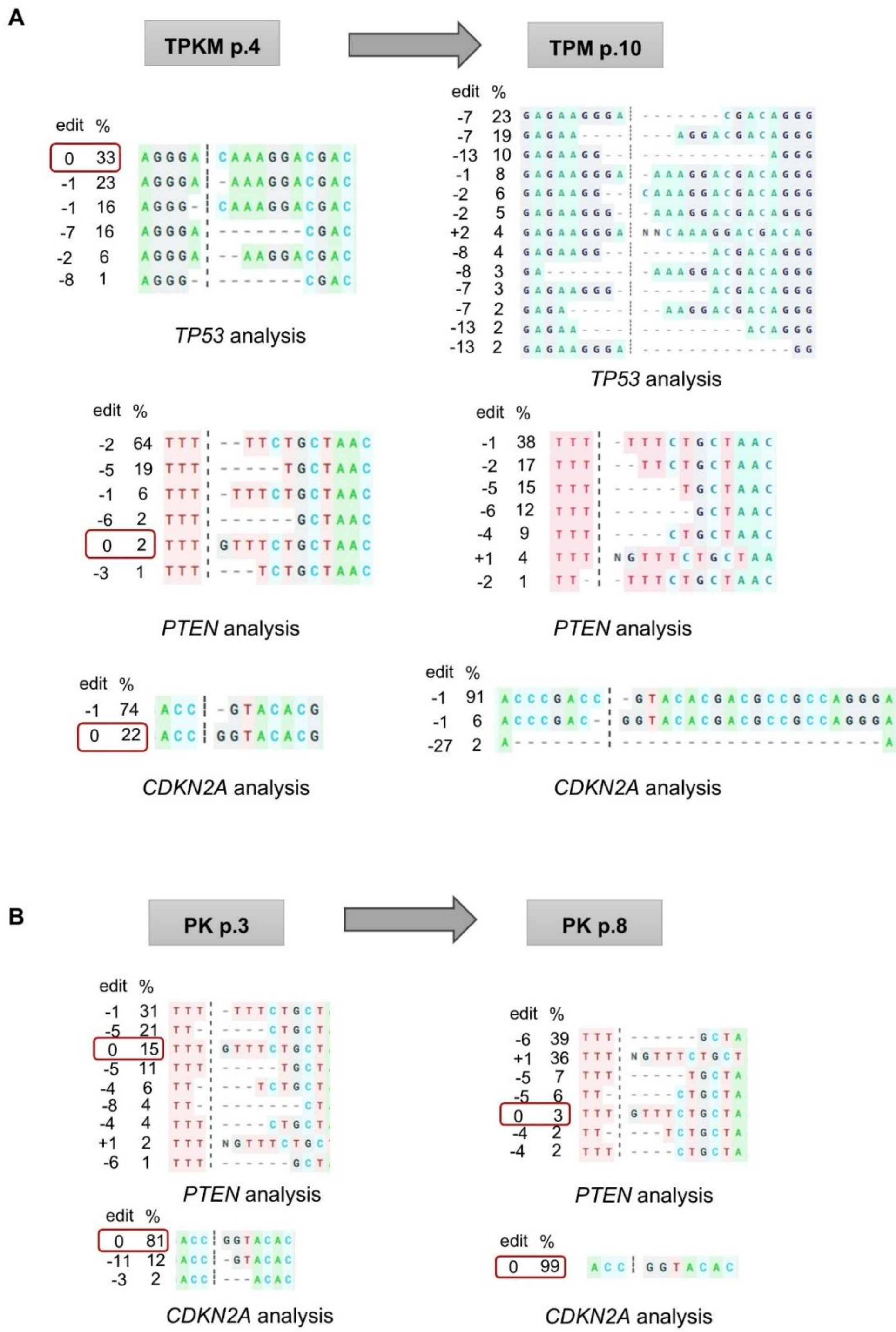


**Fig. S3. Variation of CRISPR-induced editing rates over successive passages in culture.**

A) Most cell lines showed enrichment of indels and depletion of unedited sequences in targeted tumor suppressor genes across cell passages. Representative sequencing of PG2-TPKM cells is shown, wherein unedited *TP53*, *PTEN*, and *CDKN2A* sequences were detectable at passage 4 but undetectable by passage 10, at which point all the three targeted genes contained indels. B) The only exception was the PK cell line,

which did not retain the induced indels in *CDKN2A* gene during culture, with all edited *CDKN2A* sequences being lost at later passages.

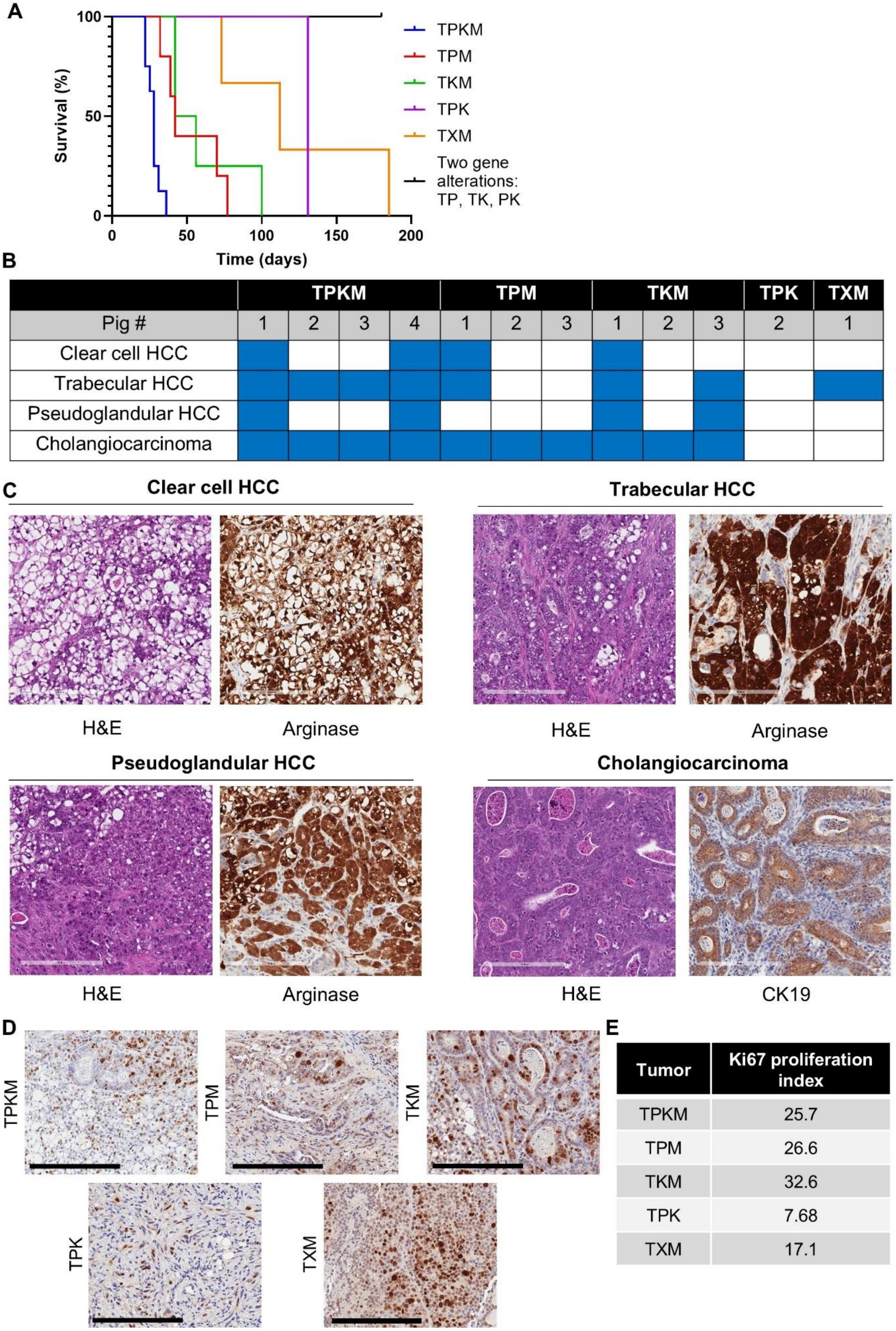


| Tumor | Ki67 proliferation index |
|---|---|
| TPKM | 25.7 |
| TPM | 26.6 |
| TKM | 32.6 |
| TPK | 7.68 |
| TXM | 17.1 |

**Fig. S4. Histopathological characterization of xenograft tumors developed by subcutaneous injection of transformed hepatocytes into SCID mice (related to Fig. 2).**

A) Kaplan–Meier survival curves of mice injected with the indicated cells. B) Heatmap indicating the presence of HCC or cholangiocarcinoma (CCA) histological features in xenograft tumors. Numbers indicate the pig of origin for each injected cell line. C) Representative TPKM tumor sections stained with H&E and Arginase or CK19, displaying features of clear cell HCC, trabecular HCC, pseudoglandular HCC, and CCA. Scale bar, 200 μm. D) Representative Ki67 staining in tumor sections. Scale bar, 300 μm. E) Ki67 proliferation index (%) quantified by digital image analysis of Ki67-stained tissue microarray cores using Halo software. Data are expressed as the percentage of Ki67-positive tumor cells.

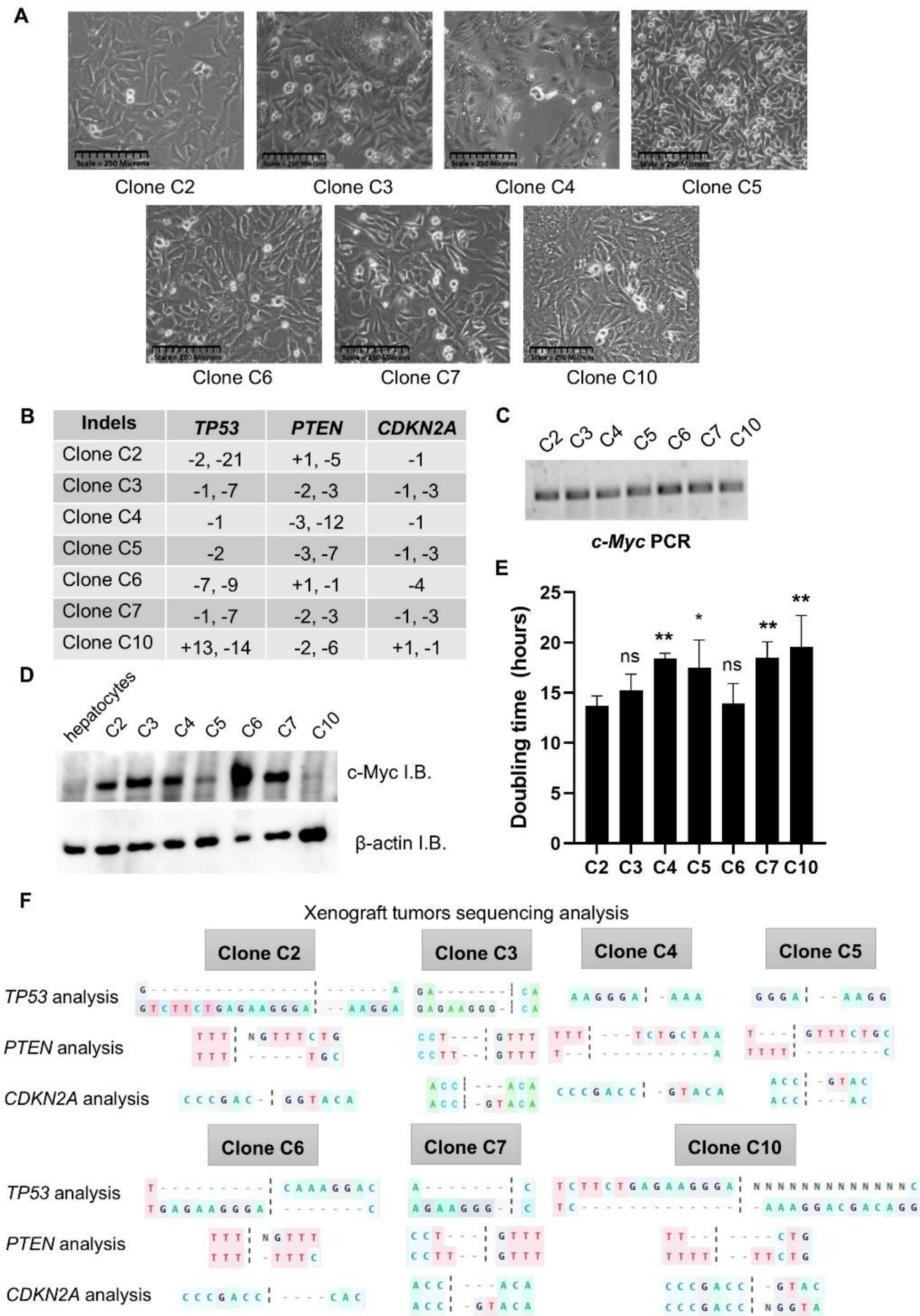


| Indels | *TP53* | *PTEN* | *CDKN2A* |
|---|---|---|---|
| Clone C2 | -2, -21 | +1, -5 | -1 |
| Clone C3 | -1, -7 | -2, -3 | -1, -3 |
| Clone C4 | -1 | -3, -12 | -1 |
| Clone C5 | -2 | -3, -7 | -1, -3 |
| Clone C6 | -7, -9 | +1, -1 | -4 |
| Clone C7 | -1, -7 | -2, -3 | -1, -3 |
| Clone C10 | +13, -14 | -2, -6 | +1, -1 |

**Fig. S5. Characterization of clonal TPKM populations and corresponding xenograft tumors.**

A) Representative pictures of seven single-cell clones derived from Pig 1 TPKM cells in culture. Scale bar, 250 μm. B) Sequencing analysis confirmed the presence of CRISPR-induced indels in the targeted genes across all clones. The type of indels in each clonal population is shown. C) PCR for genomic DNA confirms *c-Myc* integration in all clones. D) Western blotting confirms c-Myc expression in the clonal populations, with variable expression levels observed. E) Doubling time (hours) of clones are presented as mean ± SD from at least three independent measurements per clone. One-way ANOVA: ns, not significant; *, $p<0.05$; **, $p<0.01$ versus C2. F) Sequencing analysis of the xenograft tumors confirmed retention of CRISPR-induced edits present in the corresponding parental cell lines. Dashed line, predicted Cas9 cleavage position; N, nucleotide insertion; dash, deleted base.

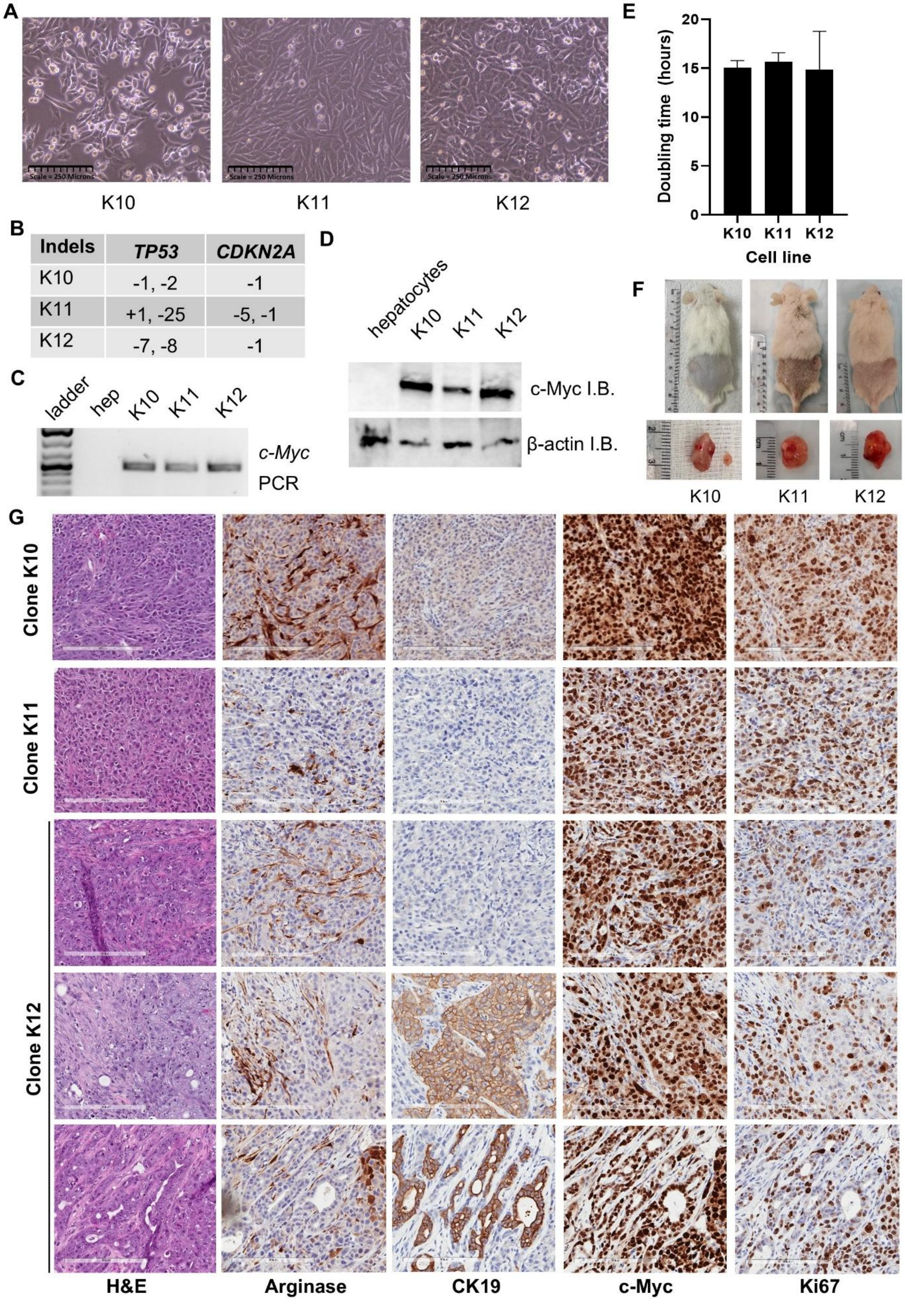


**Fig. S6. Clonal TKM cells display diverse histological features in vivo.**

A) Representative pictures of three single-cell clones isolated from Pig 1 TKM cells in culture. Scale bar, 250 µm. B) Sequencing analysis confirmed the presence of CRISPR-induced indels in *TP53* and *CDKN2A* across all clones. The type of indels in each clonal population is shown. C) PCR confirms *c-Myc* integration in all clones. D) Western blotting confirms c-Myc expression in the clonal populations, with variable expression levels observed. E) Doubling time (hours) of the cells are presented as mean ± SD from at least three independent measurements per clone. One-way ANOVA indicated no significant differences among the three clones. F) Subcutaneous injection of clonal populations into both flanks of SCID mice resulted in tumor development. Mice were euthanized when one tumor reached 1.5 cm in diameter or both tumors measured 2 cm in sum of diameters. Clone K10 resulted in the development of one large and one small tumor, while K11 and K12 clones resulted in the development of a single tumor in each mouse. G) Representative tumor sections stained with H&E, Arginase, CK19, c-Myc, and Ki67. Scale bar, 200 µm

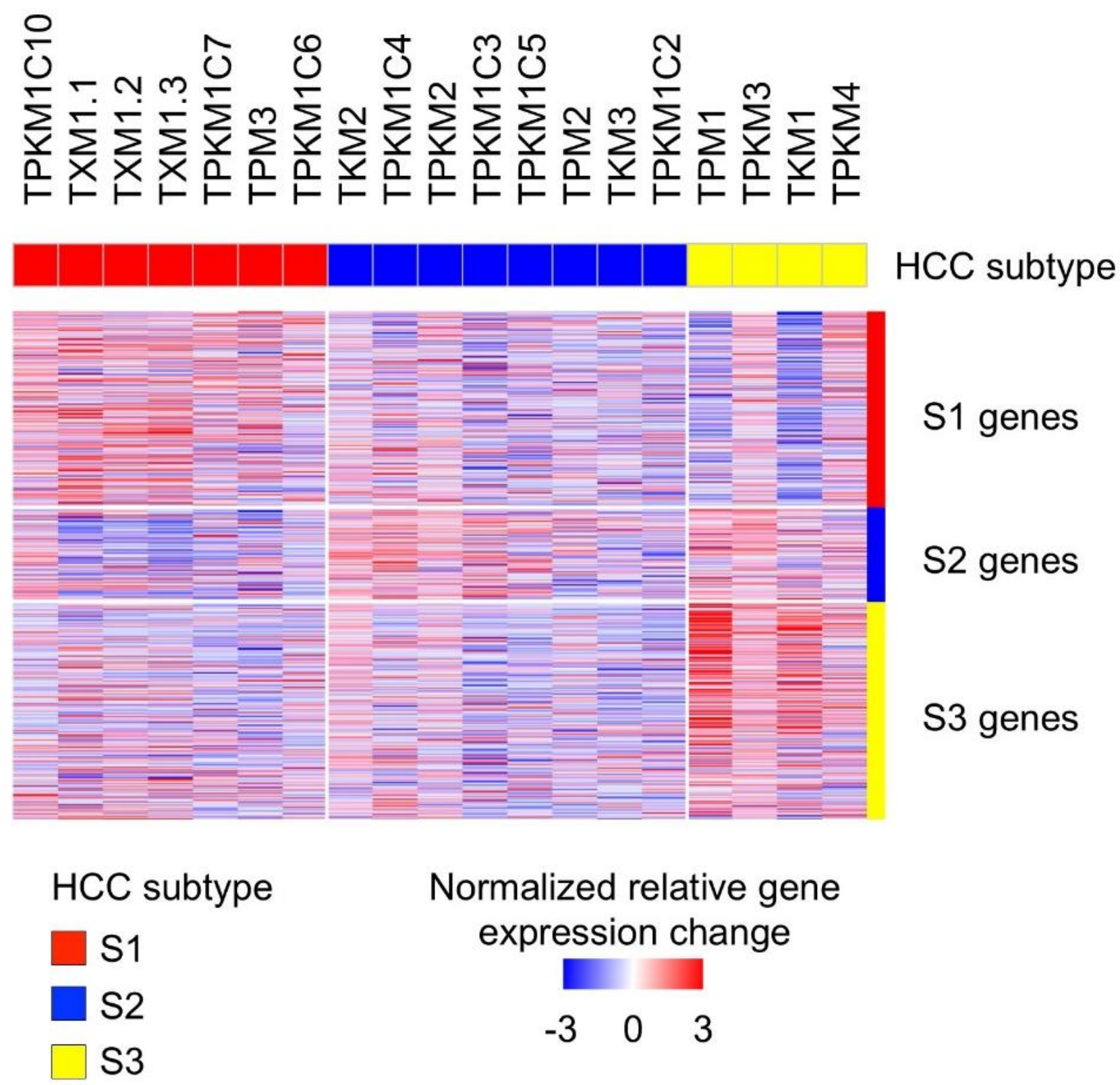


**Fig. S7. Analysis of transcriptomic meta-analysis-based human HCC subtypes in the xenograft tumors.**

Expression heatmap of the Hoshida 619-gene HCC subtype signature in TPKM, TPM, TKM, and TXM xenograft tumors. Z-score color scale is shown.

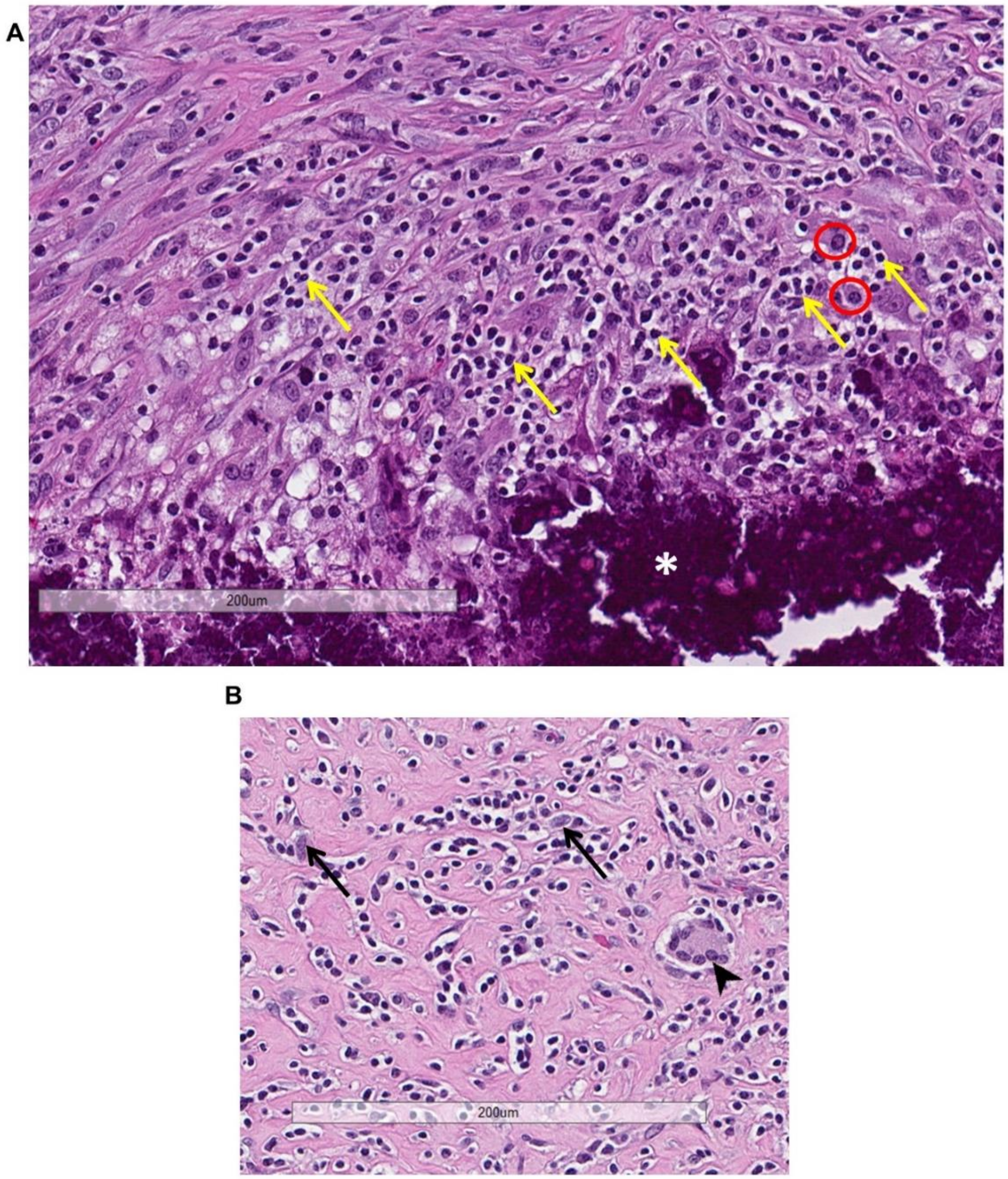


**Fig. S8. Higher-magnification H&E images demonstrating tumor immune cell infiltration in porcine liver tumors.**

A) Representative image showing lymphocytes (yellow arrows), plasma cells (red circles), and areas of calcification (white asterisk). B) Representative image showing a multinucleated giant cell (arrow head) and histiocytes (black arrows). Images are higher-magnification views of the tumor shown in **Fig. 6D**. Scale bar, 200 μm.

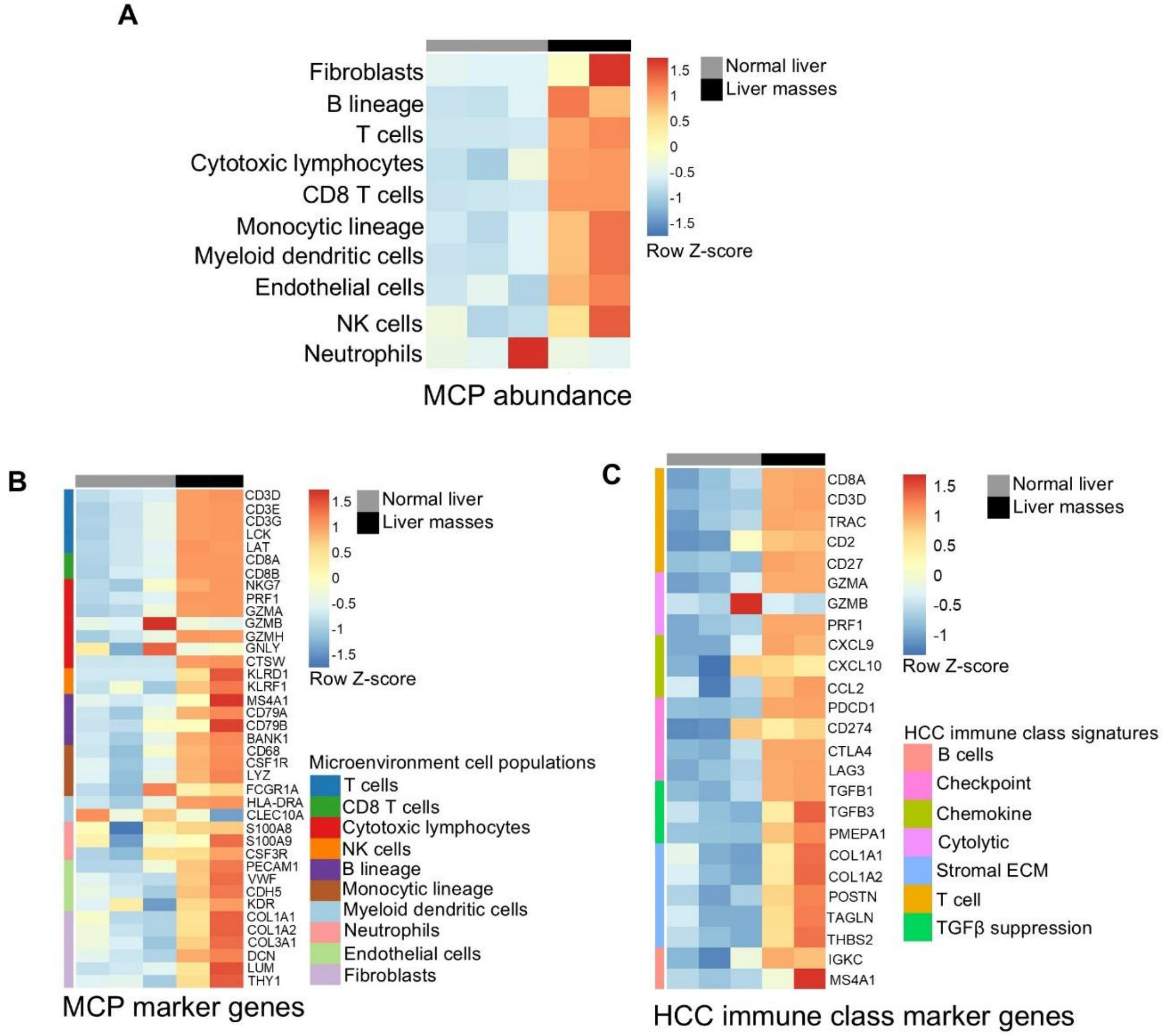


**Fig. S9. RNA sequencing-based profiling of the tumor microenvironment in porcine HCC tumors.**

A) Heatmap of row-scaled MCP-counter scores showing the relative abundance of immune and stromal cell populations across normal liver and pig tumors. B) Heatmap of MCP-counter marker gene expression displayed as row Z-scores of log2-transformed normalized counts. C) Heatmap showing relative expression of HCC immune class signature genes across normal liver and pig tumors. Row Z-scores are shown.
