# Supplementary material for: Modeling liver cancer molecular and histological features by CRISPR editing of porcine hepatocytes
Source: JHEP Rep. 2026 Sep 23;8(12):102042. doi: 10.1016/j.jhepr.2026.102042 (PMC13770662; doi:10.1016/j.jhepr.2026.102042)
Supplement: Supplementary file 3 [file mmc5.pdf]

JHEP|Reports

# Modeling liver cancer molecular and histological features by CRISPR editing of porcine hepatocytes

## Authors

Lobna Elkhadragy, Olayinka G. David, Caitlyn C. Castillo, …, Grace Guzman, Kyle M. Schachtschneider, Ron C. Gaba

## Correspondence

lobna@uic.edu (L. Elkhadragy).

## Graphical abstract

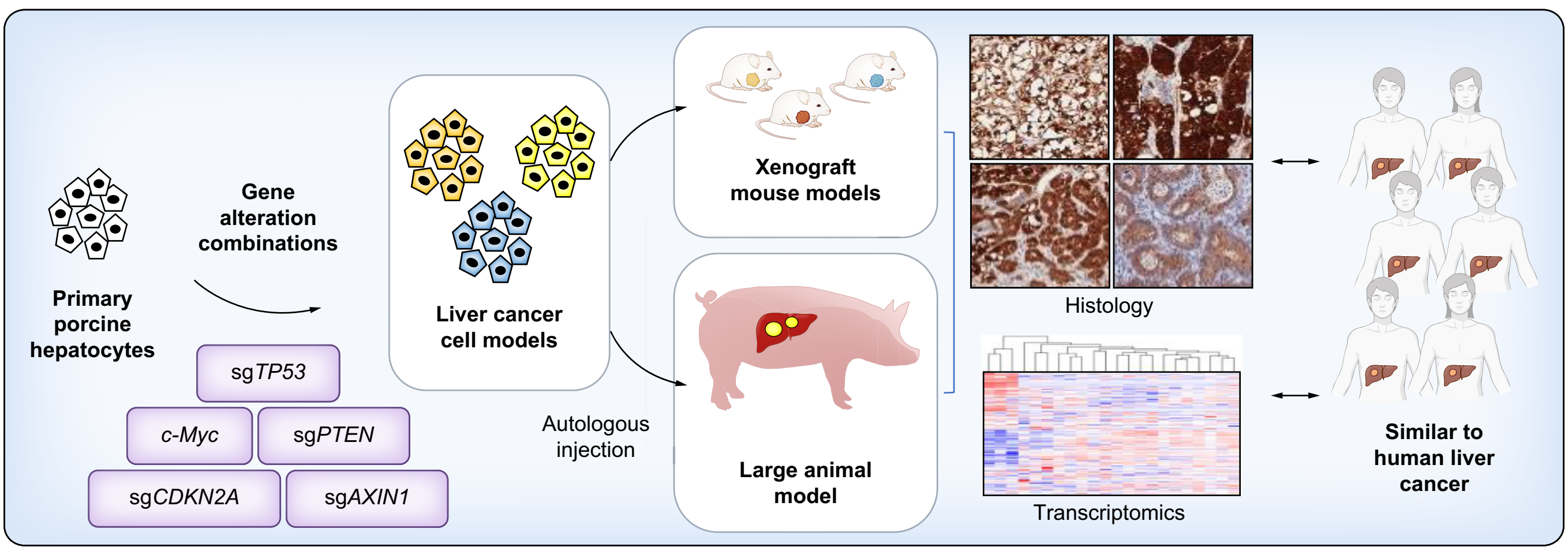


## Highlights:

- *Ex vivo* CRISPR editing of porcine hepatocytes generates liver cancer cell models.
- Three or more gene alterations are required for hepatocyte transformation.
- c-Myc cooperates with tumor suppressor loss to drive hepatocarcinogenesis.
- Generated tumors recapitulate key molecular and histological features of human HCC.
- Autologous implantation establishes proof of concept for a novel porcine HCC model.

## Impact and implications:

Hepatocellular carcinoma (HCC) shows limited responses to available treatments, underscoring the urgent need for translational preclinical models. Through *ex vivo* CRISPR editing of clinically relevant genes in porcine hepatocytes, we established cell and animal models that recapitulate key molecular and histological features of human HCC. This platform provides a foundation for future mechanistic studies, genotype-phenotype mapping, and preclinical testing of precision therapies. Moreover, the porcine model's size and physiological similarity to humans make it a promising model for evaluating locoregional therapies and device-based therapies in an immunocompetent setting.

https://doi.org/10.1016/j.jhepr.2026.102042

© 2026 The Author(s). Published by Elsevier B.V. on behalf of European Association for the Study of the Liver (EASL). This is an open access article under the CC BY license (http://creativecommons.org/licenses/by/4.0/). 

# Modeling liver cancer molecular and histological features by CRISPR editing of porcine hepatocytes

Lobna Elkhadragy[1,*], Olayinka G. David[1], Caitlyn C. Castillo[1], Luke N. Redlon[1], Luke R. Jordan[1], Nusrat I. Khan[1], Hannah Liu[1], Isadora Andre Rosa Lopes[1], Yanqiong Zhou[1], Hiroaki Kanzaki[2], Yujin Hoshida[2], Jonathan P. Samuelson[3], Amaia Lujambio[4], Lawrence B. Schook[1,5], Grace Guzman[6], Kyle M. Schachtschneider[1], Ron C. Gaba[1,6]

JHEP Reports **2026**. vol. 8 | 1–15

Check for updates

**Background & Aims:** Hepatocellular carcinoma (HCC) is an aggressive disease for which effective therapeutic options remain limited. Progress in therapeutic development has been hampered by the lack of preclinical models that recapitulate human HCC and support the evaluation of multimodal therapies. Here, we employed CRISPR editing to identify alterations that drive hepatocyte transformation and establish genetically defined HCC models.

**Methods:** Hepatocytes were isolated from pigs (n = 7) and subjected to combinations of 2-4 gene alterations, including CRISPR-mediated knockout of tumor suppressor genes (*TP53*, *PTEN*, *CDKN2A*, *AXIN1*), with or without *c-Myc* overexpression. Resulting cells were injected subcutaneously into immunodeficient mice to assess their tumorigenicity and histological features. To establish a large-animal model, autologous transformed hepatocytes were implanted at four intrahepatic sites in pigs (n = 5). Transcriptomic profiling of cells, xenografts, and porcine tumors was conducted by RNA sequencing, using FDR <0.05 for differential gene expression analysis.

**Results:** Eight combinations of gene alterations yielded cells that could be expanded in culture, in contrast to unmodified primary hepatocytes. Cells harboring at least three gene alterations (n = 5 lines) formed xenograft tumors (n = 22 mice), whereas cells with only two gene knockouts did not (n = 5 mice). Xenografts with sg*TP53*; *c-Myc*$^{OE}$; sg*PTEN* and/or sg*CDKN2A* recapitulated histological features of human liver cancer, including clear cell, trabecular, and pseudoglandular HCC and cholangiocarcinoma. Transcriptomic analysis of transformed cells and xenograft tumors revealed activation of cell-cycle pathways and similarities to human HCC profiles. Autologous implantation of transformed hepatocytes generated intrahepatic tumors at 7/8 injection sites (87.5%) in two pigs, providing proof of concept for an immunocompetent HCC model in wild-type pigs.

**Conclusions:** This study delineates cooperative oncogenic alterations that drive hepatocyte transformation *ex vivo* and provides cell and animal models that recapitulate key histological and molecular features of human HCC.

© 2026 The Author(s). Published by Elsevier B.V. on behalf of European Association for the Study of the Liver (EASL). This is an open access article under the CC BY license (http://creativecommons.org/licenses/by/4.0/).

## Introduction

Liver cancer is a leading cause of cancer-related mortality worldwide, accounting for more than 830,000 deaths annually.[1] The most common type of primary liver cancer is hepatocellular carcinoma (HCC), representing 85-90% of cases, followed by cholangiocarcinoma.[2] Despite recent advances, therapies for unresectable HCC offer only modest survival benefits,[2] highlighting the need for more effective treatment strategies. Progress in therapeutic development has been hindered by the limited availability of physiologically relevant preclinical models that enable the study of HCC initiation and the evaluation of multimodal therapies.

HCC arises through the accumulation of genetic alterations, with hepatocytes considered the most likely cell of origin.[2] Although the mutational landscape of HCC has been extensively characterized by sequencing studies,[3–5] the causal roles of distinct gene alterations in malignant transformation remain poorly defined. Introducing gene alterations into normal hepatocytes can identify the oncogenic events required to initiate HCC. Prior *in vitro* hepatocyte transformation studies relied on overexpression of oncogenes such as *SV40* and *HRAS* in hepatocytes or fibroblasts reprogrammed into induced hepatocytes.[6–9] However, SV40 is an experimental viral oncoprotein, *HRAS* mutations are rare in human HCC, and induced hepatocytes do not fully mimic mature hepatocyte biology, limiting the translational relevance of these systems. To date, transformation of hepatocytes into HCC through *ex vivo* gene editing has not been demonstrated.

Another major barrier to therapeutic advancement is the lack of preclinical models that faithfully recapitulate human HCC and support the evaluation of clinically relevant interventions. Although murine models have yielded important biological insights, few drug candidates that show promise in mice ultimately prove safe and effective in clinical trials, owing in large part to substantial physiological differences between mice and

* Corresponding author. Address: Department of Radiology, University of Illinois Chicago, 2201 W Campbell Park Drive, Suite 210, Chicago, IL 60612, USA.
*E-mail address:* lobna@uic.edu (L. Elkhadragy).
https://doi.org/10.1016/j.jhepr.2026.102042

humans.[10,11] Furthermore, the small size of mice makes them unsuitable for evaluation of devices and locoregional therapies (LRTs) such as embolization and ablation, which are used in more than half of patients.[12–14] Pigs are a valuable disease model because of their close similarity to humans in genetics, physiology, immunity, and drug metabolism.[15] A porcine liver cancer model recently developed using genetically engineered Oncopigs has proven useful for testing LRTs.[16–18] However, its reliance on $KRAS^{G12D}$, a mutation rarely observed in human HCC, limits its translational relevance.

To address these gaps, we used *ex vivo* CRISPR editing of porcine hepatocytes to identify combinations of gene alterations that induce transformation. Injection of these edited cells into SCID mice resulted in xenograft tumor development, and autologous intrahepatic implantation in pigs established an immunocompetent large-animal model. These preclinical models recapitulated key histological and transcriptional features of human HCC, providing a physiologically relevant platform for studying tumor initiation and evaluating precision and multimodal therapies.

## Materials and methods

### Animals

All procedures were conducted under protocols approved by the University of Illinois Institutional Animal Care and Use Committee. Seven male Yucatan Minipigs (Premier BioSource) were used (Table S1). Nine-to 12-week-old male severe combined immunodeficiency (SCID) mice (NOD.CB17-$Prkdc^{scid}$/J, Jackson Laboratory #001303) were used to assess cell tumorigenicity and tumor histological features.

### Hepatocyte isolation

A liver section was resected from each pig and used for isolation of hepatocytes by collagenase digestion as previously described.[19] Pig 1 was euthanized followed by immediate partial liver resection, whereas Pigs 2-7 were anesthetized and underwent partial liver resection via survival surgery (Table S1). Isolated hepatocytes were suspended in hepatocyte plating medium (Lonza #MP250) and seeded in Primaria plates (Corning #353847).

### CRISPR/Cas9 gene editing

One day post-seeding, the hepatocytes were transfected with 25 nM CRISPR ribonucleoprotein (RNP) complexes using Lipofectamine CRISPRMAX transfection reagent (Invitrogen #CMAX00003), and the medium was switched to hepatocyte maintenance medium (Gibco #A1217601, CM4000). Each RNP complex was formed by combining purified *S. pyogenes* Cas9 nuclease (IDT #1081060) with a single-guide RNA (sgRNA) as previously described.[19] The sgRNAs were generated by incubating equimolar ratios of CRISPR RNA (crRNA; sequences in Table S2, synthesized by IDT) and trans-activating CRISPR RNA (tracrRNA; IDT #1072532) at 95°C for 5 minutes. sgRNAs targeting porcine *PTEN*, *CDKN2A*, and *AXIN1* were previously validated.[20,21] For *TP53*, two sgRNAs were designed using CRISPOR and screened in a previously established porcine cell line.[21] The sgRNA demonstrating higher gene editing efficiency (*TP53* sgRNA#1) was selected for subsequent experiments (Fig. S1).

### Lentiviral vector transduction

Two days post-transfection with CRISPR RNPs, hepatocytes (for selected conditions) were transduced with *c-Myc* lentiviral vector in the presence of 6 µg/ml polybrene (Santa Cruz Biotechnology #sc-134220) and 50 µM vitamin E. Lentivirus was produced by cotransfecting Lenti-X™ 293T cells (Takara Bio #632180) with pCDH-*Myc*-Puro (Addgene #46970), psPAX2 packaging plasmid (Addgene #12260), and pMD2.G envelope-coding plasmid (Addgene #12259) using polyethylenimine (Polysciences #24765). Viral supernatant was harvested at 48 and 72 hours, and concentrated using PEG-it Virus Precipitation Solution (System Biosciences #LV825A). Lentiviral vectors were then resuspended in PBS containing calcium and magnesium, aliquoted, and stored at -80°C.

### Cell line propagation

Following CRISPR RNP transfection (with or without lentiviral transduction), medium was replaced every 2-3 days with fresh hepatocyte maintenance medium. On day 10 post-seeding, the medium was replaced with DMEM supplemented with 10% FBS and 1% penicillin-streptomycin (Gibco). Cells were passaged 2-3 days later and subsequently passaged at 80-95% confluence. Cell lines were designated according to the induced gene alterations, with each alteration represented by a letter abbreviation as follows: sgTP53 (T), sgPTEN (P), sgCDKN2A (K), sgAXIN1 (X), and *c-Myc* overexpression (M) (Fig. 1A,B). All the generated cell lines were maintained in culture for at least 30 passages.

### Analysis of gene alterations

Genomic DNA was extracted from cells at different passages using QuickExtract DNA Extraction Solution (Lucigen #QE09050).[19] *c-Myc* integration was confirmed by PCR with primers listed in Table S2, followed by agarose gel electrophoresis. For CRISPR editing analysis, the genomic locus flanking each Cas9 target site was amplified by PCR for 28 cycles using primers listed in Table S2. PCR products were subjected to Sanger sequencing and gene editing efficiency was quantified using Inference of CRISPR Edits (ICE) software (ice.synthego.com), which quantifies the frequency of sequences containing insertions and deletions (indels).

### Subcutaneous mouse injections

SCID mice were anesthetized with isoflurane, and both flanks were shaved and wiped with alcohol. Cells ($5 \times 10^6$) suspended in 90 µl PBS were mixed with an equal volume of hydrogel (TheWell Bioscience #VHM01) and injected subcutaneously into each flank with a 20-gauge needle. Tumor size was monitored by caliper measurements twice a week for a total of 6 months. Mice were euthanized when one tumor reached 1.5 cm in diameter or both tumors measured 2 cm in sum of diameters. Time-to-euthanasia results were presented as mean ± SD and analyzed using Kruskal–Wallis test with Dunn's multiple comparisons. Tumors were harvested and dissected, with one portion fixed in 10% formalin for histologic processing, and the remaining portion flash-frozen in liquid nitrogen followed by storage at

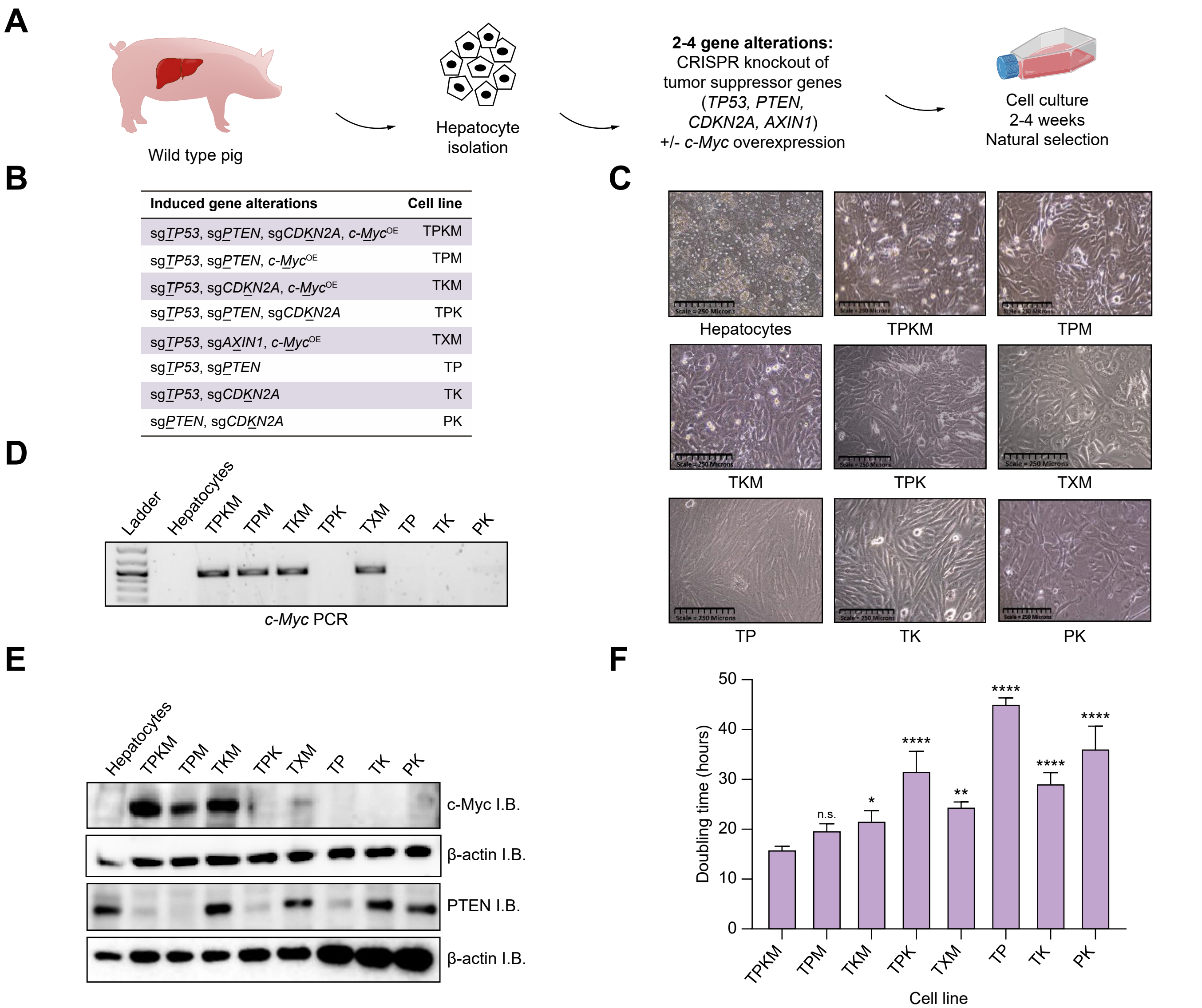


| Induced gene alterations | Cell line |
|---|---|
| sg*TP53*, sg*PTEN*, sg*CDKN2A*, *c-Myc*$^{OE}$ | TPKM |
| sg*TP53*, sg*PTEN*, *c-Myc*$^{OE}$ | TPM |
| sg*TP53*, sg*CDKN2A*, *c-Myc*$^{OE}$ | TKM |
| sg*TP53*, sg*PTEN*, sg*CDKN2A* | TPK |
| sg*TP53*, sg*AXIN1*, *c-Myc*$^{OE}$ | TXM |
| sg*TP53*, sg*PTEN* | TP |
| sg*TP53*, sg*CDKN2A* | TK |
| sg*PTEN*, sg*CDKN2A* | PK |

**Fig. 1. Development of expandable cell populations by *ex vivo* CRISPR editing of porcine hepatocytes.** (A) Primary hepatocytes were isolated from wild-type pig liver sections and subjected to 2-4 gene alterations, including CRISPR-mediated knockout of tumor suppressor genes, with or without *c-Myc* OE. The cells were cultured for 2-4 weeks to allow the natural selection of cells with gene alterations conferring a growth advantage. (B) Eight gene alteration combinations resulted in the development of expandable cells from one or more pigs (see Table S1). The resulting cells were designated based on the induced gene alterations. (C) Representative images of primary porcine hepatocytes (day 1 post-isolation) and established cell lines after ≥2 weeks in culture. Scale bar, 250 μm. (D) PCR confirms *c-Myc* integration in TPKM, TPM, TKM, and TXM cells. (E) Western blotting confirms c-Myc overexpression in TPKM, TPM, TKM, and TXM cells and reduced PTEN protein expression in *PTEN*-targeted cells. β-actin was used as a loading control. (F) Doubling time (hours) of the cells are presented as mean ± SD from at least three independent measurements per cell line. One-way ANOVA: n.s., not significant; *$p$ <0.05; **$p$ <0.01; ****$p$ <0.0001 *vs.* TPKM. OE, overexpression; sg, single-guide RNA.

-80°C for DNA/RNA extraction and transcriptional analysis. DNA was extracted from mouse tumors using Quick-DNA Miniprep Plus Kit (Zymo Research #D4068).

## Intrahepatic pig injections

Autologous transformed hepatocytes were injected into four distinct sites of the liver parenchyma under ultrasound guidance (n = 5 pigs, Table S1). Cells were washed with PBS, resuspended in 150-400 μl PBS, and mixed with scaffold material immediately prior to injection. In Pig 2, synthetic hydrogel (TheWell Bioscience #VHM01) was used as a scaffold at all injection sites. In Pigs 3 and 4, cells injected into sites 1 and 2 were mixed with a slurry of gelatin sponge fragments (Surgifoam; Ethicon #1972) by gentle repeated passage through a 3-way stopcock, while cells injected into sites 3 and 4 were mixed with an equal volume of synthetic hydrogel (TheWell Bioscience #VHM01). In Pigs 6 and 7, Matrigel (Corning #354234) was used as a scaffold at two injection sites and VitroGel® RGD (TheWell Bioscience #TWG003) was used at two sites. Injections were performed using a 16-gauge 4-inch needle (AirTite #N164). Pigs 3, 4, 6, and 7 received oral

prednisolone (1 mg/kg) in food treats starting one day before injections, on the day of injections, and for three additional days after cell injection.

### Imaging and harvesting of pig intrahepatic tumors

Tumor growth was monitored by ultrasound or contrast-enhanced CT at 1–3-week intervals. CT protocols included non-contrast, arterial phase at 30-35 seconds, portal venous phase at 60-70 seconds, and delayed phase at 180 seconds after 120-150 ml of iodinated contrast was injected intravenously at a rate of 4-5 ml/s. Intrahepatic masses harvested at euthanasia were transected and one half was stored in 10% formalin for histologic processing and the other half was flash frozen in liquid nitrogen and stored at -80°C for transcriptional analysis.

### RNA extraction

Total RNA was extracted from cultured cells, mouse xenograft tumors, pig liver, and porcine intrahepatic masses using TRIzol Reagent (Invitrogen #15596018) followed by purification with Direct-zol RNA Miniprep Plus kit (Zymo Research #R2070), including on-column DNase treatment. Cells analyzed included TPKM cells derived from pigs 1-4; TPM and TKM cells from pigs 1-3, TPK cells from pig 2, TXM cells from pig 1, and four single-cell clones isolated from TPKM (C2, C4, C5, and C10). Unmodified hepatocytes from pigs 3-5 served as controls. Xenografts analyzed included TPKM tumors derived from pigs 1-4, TPM and TKM tumors derived from pigs 1-3, TXM tumors derived from pig 1, and tumors developed from six TPKM single cell clones (C2, C4, C5, C6, C7, and C10). Four intrahepatic masses from pig 3 were analyzed. Normal porcine liver tissue from pigs 2, 3, and 5 served as controls for xenograft and pig tumors.

### Transcriptional analysis

RNA-seq libraries were prepared using the Kapa mRNA HyperPrep kit (Roche #KK8581) at the University of Illinois Urbana-Champaign Biotechnology Center. Libraries were quantitated by qPCR and sequenced on an Illumina NovaSeq X Plus (v1.0 sequencing kits) generating 151-bp paired-end reads at a depth of ≥25 million reads per sample. TXM samples were sequenced in a separate batch and processed using the same bioinformatic pipeline as the original dataset. Paired-end reads were quality-checked with FASTQC v0.12.0 and adapter-trimmed using Trimmomatic v0.36. Reads shorter than 36 bases after trimming were discarded. Trimmed paired-end reads were aligned to the *Sus scrofa* reference genome (Sscrofa11.1, Ensembl release 112) using STAR v2.7.11b in two-pass mode. Transcript abundance was estimated using RSEM v1.3.3, and porcine-human ortholog mapping was performed using the BioMart library. Differentially expressed gene (DEG) analysis, principal component analysis (PCA), hierarchical clustering heatmaps, and volcano plots were generated with DEBrowser v1.34.0. Ingenuity Pathway Analysis (IPA) software was used for pathway analysis. Transcriptomic meta-analysis-based human HCC subtypes (Hoshida's S1/S2/S3 subtypes) were determined in xenografts and porcine intrahepatic tumors based on the 619 subtype signature genes as previously described using the NTP (nearest template prediction) algorithm.[22,23]

### Tumor microenvironment profiling

Tumor microenvironment composition was assessed using RNA-seq data for masses 1 and 2 from pig 3 with microenvironment cell population (MCP)-counter.[24] Log2-transformed normalized counts were used to estimate the abundance of immune and stromal cell populations. MCP-counter scores and expression levels of associated signature genes were visualized as row-scaled heatmaps in R. Differences between tumor and normal liver tissue were assessed using the Wilcoxon rank-sum test with Benjamini–Hochberg correction for multiple testing. In addition, expression of previously described HCC immune class signature genes was evaluated in pig tumors and normal liver tissue.[25]

Additional experimental procedures are provided in the Supplementary Materials and Methods section.

## Results

### CRISPR-induced gene alterations in primary porcine hepatocytes generate expandable cell populations

To uncover genetic events required for HCC initiation, we introduced defined gene alterations into primary porcine hepatocytes. Candidate genes were selected based on the mutational landscape of HCC and the combinations were chosen based on patterns of mutation co-occurrence identified in human and mouse studies.[3–5,26–29] Target genes included tumor suppressor genes frequently inactivated in human HCC (*TP53*, *PTEN*, *CDKN2A*, and *AXIN1*) and the oncogene *c-Myc*, which is often overexpressed or amplified in HCC (Fig. 1A). Loss-of-function mutations were induced in the tumor suppressor genes using CRISPR/Cas9 with sgRNAs (Table S2), while *c-Myc* overexpression (OE) was achieved by lentiviral transduction.

Primary hepatocytes were isolated from wild-type pigs and subjected to combinations of 2-4 gene alterations (Fig. 1B, Table S1). As expected,[30] unmodified control hepatocytes were not expandable in culture. In contrast, hepatocytes subjected to eight different combinations of gene alterations yielded expandable cell lines capable of serial passaging *in vitro*. Cell lines were designated according to the induced gene alterations, for example cells generated by sg*TP53*, sg*PTEN*, sg*CDKN2A*, and *c-Myc* OE were referred to as TPKM (Fig. 1B). The generated cells were maintained in culture for 2-4 weeks to allow the natural selection of cells with higher proliferative capacity (Fig. 1C).

Sequencing analysis confirmed CRISPR-mediated editing, with indels detected in all targeted genes across the generated cell lines (Fig. S2). Consistent with expected CRISPR/Cas9 outcomes, pools of cells contained a mixture of indels. In all the generated cells except for PK, there was an enrichment of cells with indels in tumor suppressor genes over successive passages (Fig. S3), indicating that the induced alterations cooperatively conferred a selective growth advantage to the cells. *c-Myc* integration in TPKM, TPM, TKM, and TXM cells was confirmed by PCR (Fig. 1D), and expression was validated by Western blotting, although TXM cells exhibited lower levels

of c-Myc compared to the other cell lines (Fig. 1E). Western blotting also confirmed reduced PTEN protein expression in *PTEN*-targeted cell lines compared with control hepatocytes (Fig. 1E).

Cell proliferation rates varied with genotype (Fig. 1F). TPKM and TPM cells proliferated fastest, whereas TKM, TPK, and TXM cells showed slower growth. Cells with only two alterations exhibited larger, flattened morphology, and significantly longer doubling times (Fig. 1C,F). These findings demonstrate that introducing distinct gene alterations into primary hepatocytes generates expandable cell populations with varying proliferative characteristics.

### Mutation combinations confer clinically relevant liver cancer phenotypes *in vivo*

We next assessed the tumorigenic potential of the generated cell lines by injecting them subcutaneously into SCID mice. Cell lines with three or four gene alterations (TPKM, TPM, TKM, TPK, and TXM) consistently formed solid pale tumors, whereas cell lines with only two gene alterations (TP, TK, and PK) did not result in tumor development within 6 months (Figs 2A,B and S4A). Among the tumorigenic lines, TPKM cells exhibited the most rapid tumor growth and the shortest time-to-euthanasia, although these values were not statistically different from TPM or TKM (Figs 2A and S4A).

Histopathological analysis was conducted to determine whether the xenograft tumors recapitulated features of human HCC. Tumors were stained with H&E and immunostained for arginase (HCC/hepatocyte marker), CK19 (biliary/stem cell marker), c-Myc, and Ki67. Tumors derived from TPKM, TPM, and TKM cells were epithelial neoplasms composed of multiphenotypic cells with poor-to-moderate differentiation, resembling human liver cancer (Fig. 2C). Architectural patterns observed in the tumors included clear cell HCC, trabecular HCC, pseudoglandular HCC, and cholangiocarcinoma (CCA) (Figs 2C and S4B,C). HCC regions stained positive for arginase and negative for CK19, while CCA regions showed the opposite staining pattern. All neoplastic cells showed nuclear c-Myc reactivity and moderate-to-high Ki67 positivity (Figs 2C and S4D,E). In contrast, TPK tumors lacked typical HCC or CCA morphology, were arginase- and CK19-negative, and exhibited fibrotic stroma with low Ki67 staining (Figs 2C and S4D,E). TXM tumors were epithelial neoplasms resembling macrotrabecular HCC and supported by moderately dense stroma (Fig. 2C).

Together, these findings demonstrate that introducing three or four gene alterations into porcine hepatocytes drives their transformation into tumorigenic cells that generate clinically relevant HCC and CCA phenotypes, with *c-Myc* playing a critical cooperative role with *TP53*, *PTEN*, and *CDKN2A*. Given that TPKM, TPM, TKM, and TXM cells produced tumors with histological features consistent with human liver cancer, we will refer to these cells hereafter as liver cancer cells.

### Clonal liver cancer cells model features of HCC histological subtypes

To reduce the histological heterogeneity observed with pooled cell injections, we isolated single-cell clones from the TPKM and TKM pools. Seven TPKM clones were confirmed to harbor CRISPR-induced indels in *TP53*, *PTEN*, and *CDKN2A*, along with *c-Myc* integration (Figs 3A and S5A-D). The doubling times of these clones ranged from 13.7 to 19.6 hours (Fig. S5E). Injection of these clones into SCID mice resulted in the development of tumors with less histological heterogeneity compared to tumors arising from the parental cell pool (Figs 2C and 3B,C). Targeted DNA sequencing of these xenograft tumors confirmed retention of the CRISPR-induced gene alterations present in the corresponding cell lines (Fig. S5B and F). Several tumors exhibited architectural patterns consistent with human HCC (Fig. 3C), including compact HCC (clone C2) and trabecular HCC (clone C5). Some clones exhibited squamous metaplasia, including clone C6 which displayed HCC with squamous differentiation and clone C4 resembling CCA with squamous differentiation. Clone C3 displayed characteristics of the HCC sarcomatoid variant, clone C7 developed tumors exhibiting CCA, sarcomatous spindle cells, and HCC phenotypes, while clone C10 resembled CCA or CK19-positive HCC, an aggressive HCC subtype with poor outcomes.[31]

Three single-cell clones were isolated from the TKM cell pool (K10, K11, and K12) and were confirmed to harbor CRISPR-induced indels in *TP53* and *CDKN2A* with c-Myc expression (Fig. S6A–D). These clones proliferated rapidly, with doubling times of 14.9 to 17.4 hours (Fig. S6E). While all three clones formed tumors following subcutaneous injection into SCID mice, K10 produced tumors on both flanks, whereas K11 and K12 each formed a single tumor (Fig. S6F). Histological analysis revealed poorly differentiated carcinomas in K10 and K11 tumors, characterized by arginase positivity, absence of CK19, and nuclear c-Myc expression. In contrast, K12 tumors had regions with different phenotypes, including compact HCC with differentiated arginase-positive cells, as well as CK19-positive regions consistent with CCA or CK19-positive HCC (Fig. S6G), suggesting *in vivo* differentiation. The phenotypic heterogeneity observed among individual clones may reflect differences in CRISPR-induced indels, variation in c-Myc expression levels, and additional genetic or epigenetic changes acquired during clonal expansion.

Collectively, these results demonstrate the establishment of clonal transformed hepatocytes which yield tumors that recapitulate distinct liver cancer histological phenotypes. These clonal models provide a platform to interrogate associations between genotype and histotype and for evaluating their potential impact on therapeutic response.

### Transcriptomic profiling reveals activation of cell cycle pathways in the porcine liver cancer cell models

We next compared the transcriptional profiles of the generated cell lines with control unmodified hepatocytes using RNA sequencing. PCA showed that the cells developed from the various donor animals clustered distinctly from control hepatocytes (Fig. 4A). Notably, TPK and PK cells, which either formed non-HCC tumors or failed to form tumors in mice, clustered separately from the tumorigenic cells. Unsupervised hierarchical clustering corroborated these distinctions (Fig. 4B).

Differentially expressed gene (DEG) analysis identified numerous upregulated and downregulated genes in TPKM, TPM, and TKM cells (Fig. 4C-E, Table S3). Pathway analysis of these DEGs revealed activation of cell cycle and extracellular matrix (ECM) remodeling pathways (Fig. 4F, Table S4). Upregulated genes included key cell cycle regulators (*AURKA*, *AURKB*, *CCNB1*, *CCNA2*, *BUB1*, and *PLK1*) as well as ECM remodeling genes such as collagens, integrins, and matrix

**A**

| Cell line | No. of mice | Tumor incidence | Time to sacrifice (days) |
|---|---|---|---|
| TPKM | 8 | 16/16 | 27.5 ± 4.7 |
| TPM | 5 | 10/10 | 52 ± 20.1$^{\text{n.s.}}$ |
| TKM | 4 | 8/8 | 60 ± 27.5$^{\text{n.s.}}$ |
| TPK | 2 | 4/4 | 131 ± 0** |
| TXM | 3 | 6/6 | 123.3 ± 56.9** |
| TP | 1 | 0/2 | No tumors |
| TK | 2 | 0/4 | No tumors |
| PK | 2 | 0/4 | No tumors |

**B**

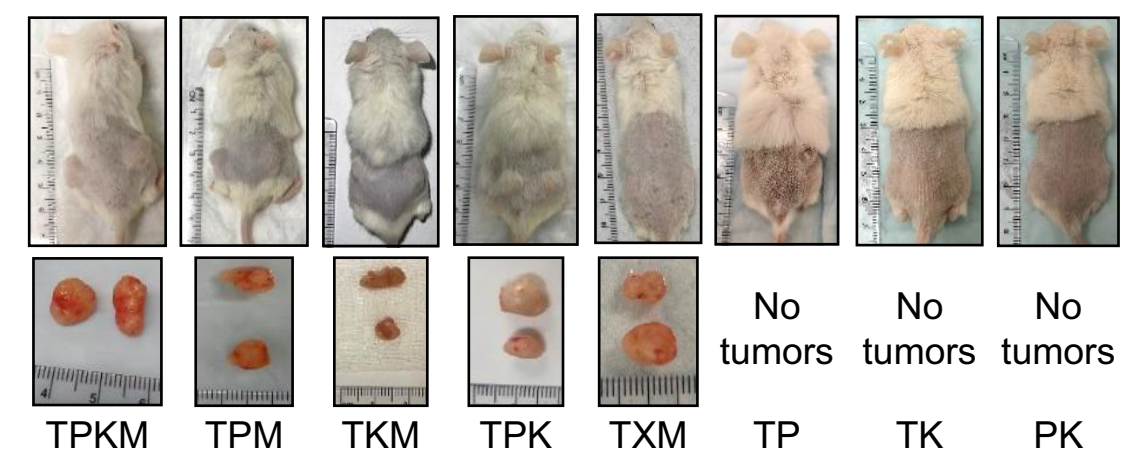


**C**

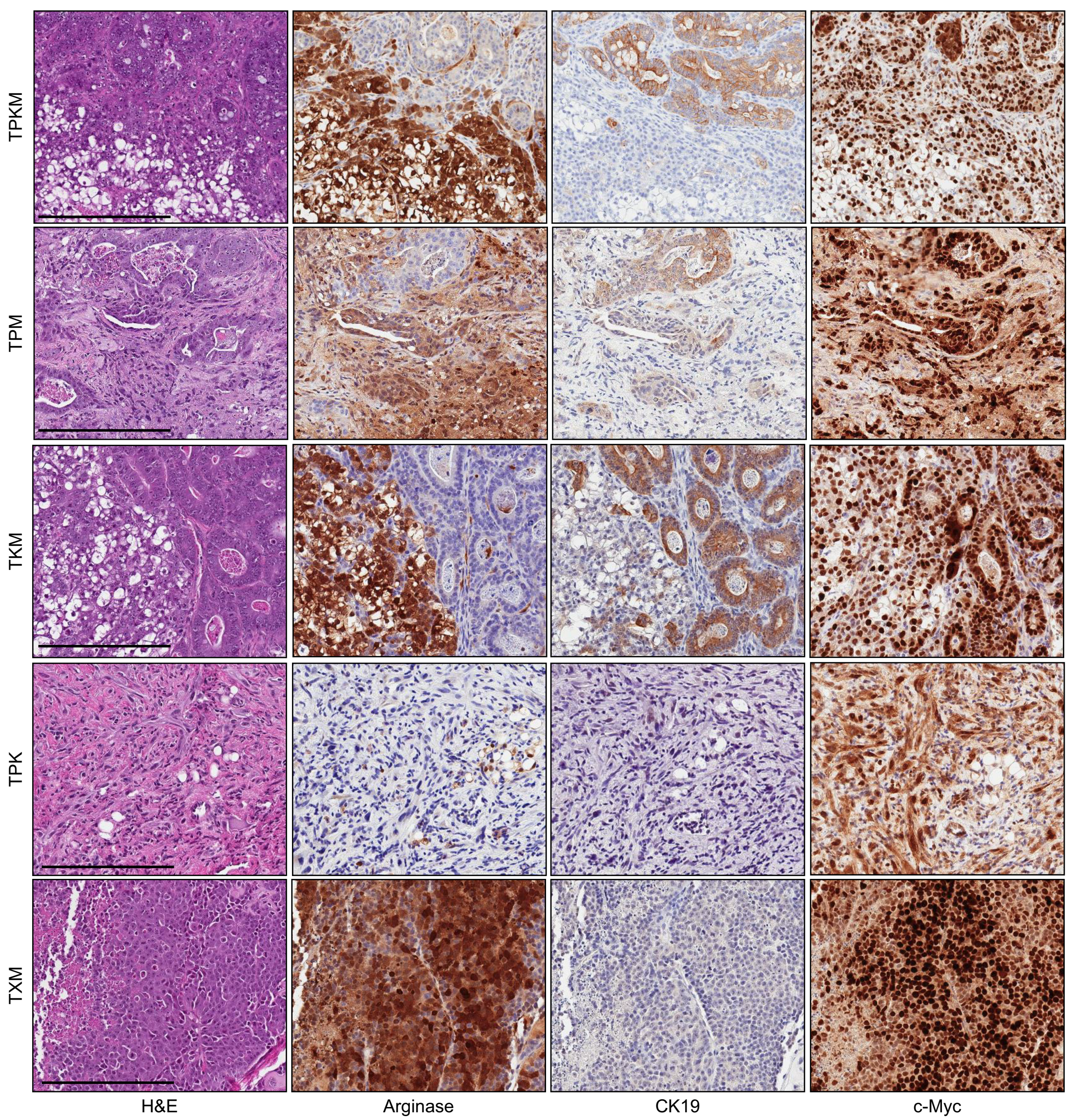


**Fig. 2. Gene alteration combinations confer clinically-relevant liver cancer phenotypes *in vivo*, including HCC and CCA.** (A) Edited cells were injected subcutaneously into both flanks of SCID mice. Mice were euthanized when one tumor reached 1.5 cm in diameter or both tumors measured 2 cm in sum of diameters. Shown are the number of mice injected, tumor incidence at the injection sites, and time to euthanize (days; mean ± SD). The porcine origin of injected cell lines is shown in Table S1 and Fig. S4B. Kruskal–Wallis test with Dunn's multiple comparisons: n.s., not significant; $^{**}p$ <0.01 *vs*. TPKM. (B) Representative pictures of shaved mice and harvested tumors. A centimeter ruler is shown for scale. The injection of TPKM, TPM, TKM, TPK, and TXM cells resulted in tumor development, whereas TP, TK, and PK cells did not within 6 months post-injection. (C) Representative tumor sections stained with H&E, arginase, CK19, and c-Myc. Scale bar, 300 µm. Ki67 staining of the same sections is shown in Fig. S4D. CCA, cholangiocarcinoma; HCC, hepatocellular carcinoma; SCID, severe combined immunodeficiency.

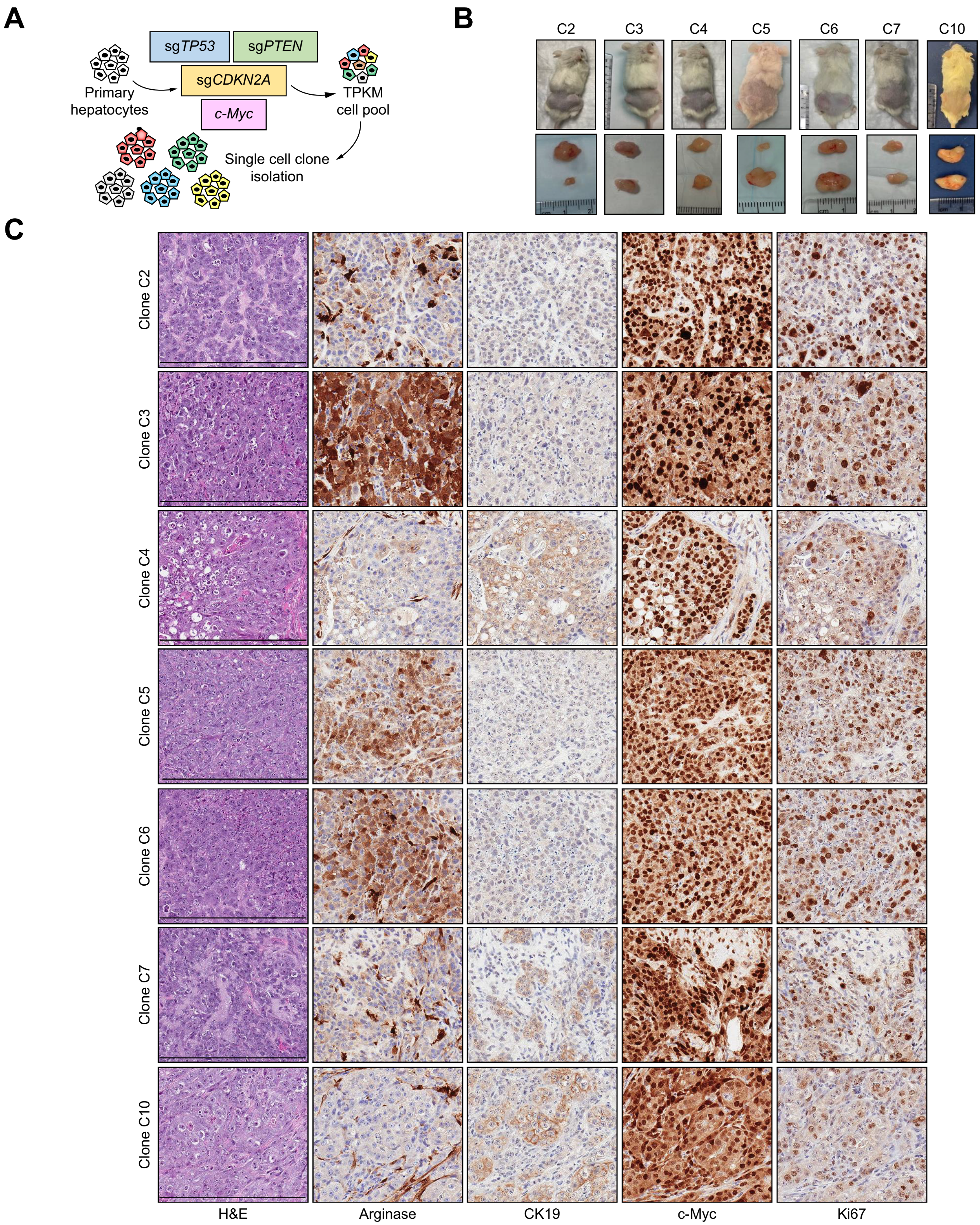


**Fig. 3. Clonal TPKM cells recapitulate diverse liver cancer histological features *in vivo*.** (A) Single cell clones were isolated from TPKM cell pool. Confirmation of gene alterations and doubling time analysis are shown in Fig. S5. (B) Subcutaneous injection of seven TPKM clones into SCID mice resulted in tumor development at all injection sites. Mice were euthanized when one tumor reached 1.5 cm in diameter or both tumors measured 2 cm in sum of diameters. Pictures of shaved mice and harvested tumors are shown. (C) Representative tumor sections stained with H&E, arginase, CK19, c-Myc, and Ki67. Scale bar, 300 µm. SCID, severe combined immunodeficiency; sg, single-guide RNA.

metalloproteinases. Alpha-fetoprotein (AFP), a biomarker of human HCC, was also highly expressed in TPKM and TKM cells. Conversely, downregulated genes were enriched in pathways related to normal liver functions, including hepatic transcription factors (*FOXA3*, *CEBPD*), Wnt pathway genes (*GLUL*, *LECT2*, *WNT2*, *WNT5A*, *WNT9A*), and drug metabolism genes (*CYP1A2*, *CYP3A4*, and *CYP2E1*).

Next, we compared the transcriptional profiles of the developed liver cancer cells TPKM, TPM, and TKM with human HCC datasets. Key pathways frequently dysregulated in human HCC, including cell cycle and Aurora B pathways[32,33] were similarly activated in all three cell models (Fig. 4F, Table S4). Additionally, the porcine liver cancer cells exhibited elevated expression of several genes frequently overexpressed in human HCC and associated with poor prognosis,[32,33] including *BUB1B*, *CDC20*, *CDCA5*, *MCM6*, *MCM3*, *TARBP1*, and *TOP2A* (Table S3). Together, these findings reveal a distinct transcriptomic profile in TPKM, TPM, and TKM cells, marked by the activation of mitosis, cell cycle, and ECM remodeling, alongside downregulation of normal liver function pathways.

### Porcine liver cancer xenografts recapitulate transcriptional features of human HCC

RNA sequencing of the xenograft tumors revealed consistent pathway alterations similar to those observed in the corresponding cell lines (Figs 4F and 5D; Tables S4 and S6). PCA and unsupervised gene expression clustering demonstrated that TPKM, TPM, TKM, and TXM xenograft tumors clustered separately from normal control porcine liver (Fig. 5A,B). Analysis of DEGs (Fig. 5C, Table S5) and altered pathways (Fig. 5D, Table S6) in these tumors identified upregulation of genes involved in cell cycle and DNA replication, including cell cycle regulators (*CDK1*, *CCNA2*, *CCNB1*, *CCNB2*), checkpoint regulators (*AURKA*, *AURKB*, *BUB1*, *CDC20*), and replication machinery (*MCM* family). *AFP* was upregulated in both TPKM and TKM tumors (Table S5), consistent with the expression pattern in the cells (Table S3). Pathways related to normal liver function were downregulated across the four tumor groups, including xenobiotic metabolism (*CYP1A1*, *CYP1A2*, *CYP2E1*), cholesterol and bile acid metabolism (apolipoprotein genes, *PPARA*, *TM7SF2*, *SC5D*), and amino acid processing (*ALDH1A*, *ALDH2*, *ALDH3A2*) (Tables S5 and S6).

We then assessed the similarity between the porcine xenograft tumors (TPKM, TPM, TKM, and TXM) and human HCC. The activated pathways in these tumors were similar to those reported in human HCC, including cell cycle and DNA replication pathways. Importantly, several genes commonly upregulated in human HCC and associated with poor survival[32] were also upregulated in the xenograft tumors, including *KPNA2*, *CDK1*, *MCM3*, *MCM6*, *PRC1*, *FEN1*, *NT5DC2*, and *LRRC1* (Table S5). In addition, the tumors exhibited overexpression of a 9-gene signature (*ANLN*, *BIRC5*, *BUB1B*, *CDC20*, *CDCA5*, *CDK1*, *NCAPG*, *NEK2*, and *TOP2A*) previously validated to be related to HCC progression and prognosis based on analyses of three large human HCC datasets.[33] The transcriptomic meta-analysis-based human HCC subtypes (Hoshida's S1/S2/S3 subtypes) were represented among the xenograft tumors, further supporting the clinical relevance of these models and their potential utility for investigating inter-tumor molecular heterogeneity (Fig. S7). Collectively, these findings demonstrate that the xenograft tumors derived from porcine liver cancer cells recapitulate key transcriptional features of human HCC and show similarities to established human HCC molecular subtypes.

## Development of a porcine HCC model by orthotopic implantation of autologous cells

To establish a large-animal HCC model, we injected autologous transformed hepatocytes into pig livers. Given the outbred nature of pigs, autologous cell injections were necessary to prevent immune rejection of allogeneic cells. In the first attempt, multiple cell lines (TPKM, TPM, TKM, and PK) were injected into each of four liver sites, using 15 million cells per site. However, no tumors were detected by serial ultrasound and CT imaging over four months of monitoring, and none were found at necropsy (Fig. 6A; Tables S1 and S7).

Hence, we implemented several modifications in subsequent experiments in Pigs 3 and 4, focusing on TPKM cells, increasing the number of cells injected to 70 million and 55 million cells per site, respectively, and administering peri-injection oral glucocorticoids, which have been shown to improve implantation success in pigs.[34] Two scaffolds were used in each pig in these experiments, either porcine-derived gelatin sponge or synthetic hydrogel. With these adjustments, intrahepatic tumors developed in both pigs at 7 of 8 injection sites (87.5% success rate) within 2-3 weeks (Fig. 6A,B; Table S7). Tumors increased in size over time and measured 0.8–3.7 cm (median 1.9 cm) at euthanasia, demonstrating a clinically relevant size suitable for testing LRTs. Notably, tumors formed at gelatin sponge injection sites were consistently larger than those at hydrogel injection sites, which consisted of aggregates of smaller nodules (Fig. 6C, Table S7). Consistent with these gross tumor findings, low-magnification H&E images demonstrated that tumors developed at hydrogel injection sites consisted of small tumor nodules surrounded by normal liver tissue (Fig. 6D). No metastatic lesions were observed in either animal, except for a mass above the liver lobe in Pig 3, which was likely due to needle tract seeding.

Histologically, the intrahepatic tumors contained arginase-positive nodules surrounding central areas of calcification or necrosis (Figs 6D,E and S8). The masses exhibited fibrosis and dense inflammatory infiltration composed predominantly of lymphocytes, with eosinophils, macrophages, multinucleated giant cells, and plasma cells also present (Fig. S8). Tumor regions showed nuclear c-Myc and Ki67 staining, while CK19 staining was absent, ruling out CCA (Fig. 6E). Additional features included vascular proliferation, hypertrophic vessels, bile duct proliferation, and granuloma formation, suggestive of a reactive response to liver injury.

To further evaluate orthotopic implantation conditions, additional experiments were performed in Pigs 6 and 7 using autologous TPKM cells mixed with either Matrigel or another type of synthetic hydrogel (VitroGel® RGD). In these experiments, 30 million cells were injected per site (Fig. 6A; Table S1). No tumors were detected by serial imaging or at necropsy in either animal (Table S7). These findings suggest that successful tumor engraftment may be influenced by implantation conditions and highlight the need for systematic evaluation of orthotopic implantation protocols, including the number of injected cells and scaffold material.

Bulk RNA sequencing of the four tumors harvested from Pig 3 was performed alongside normal porcine liver controls. PCA

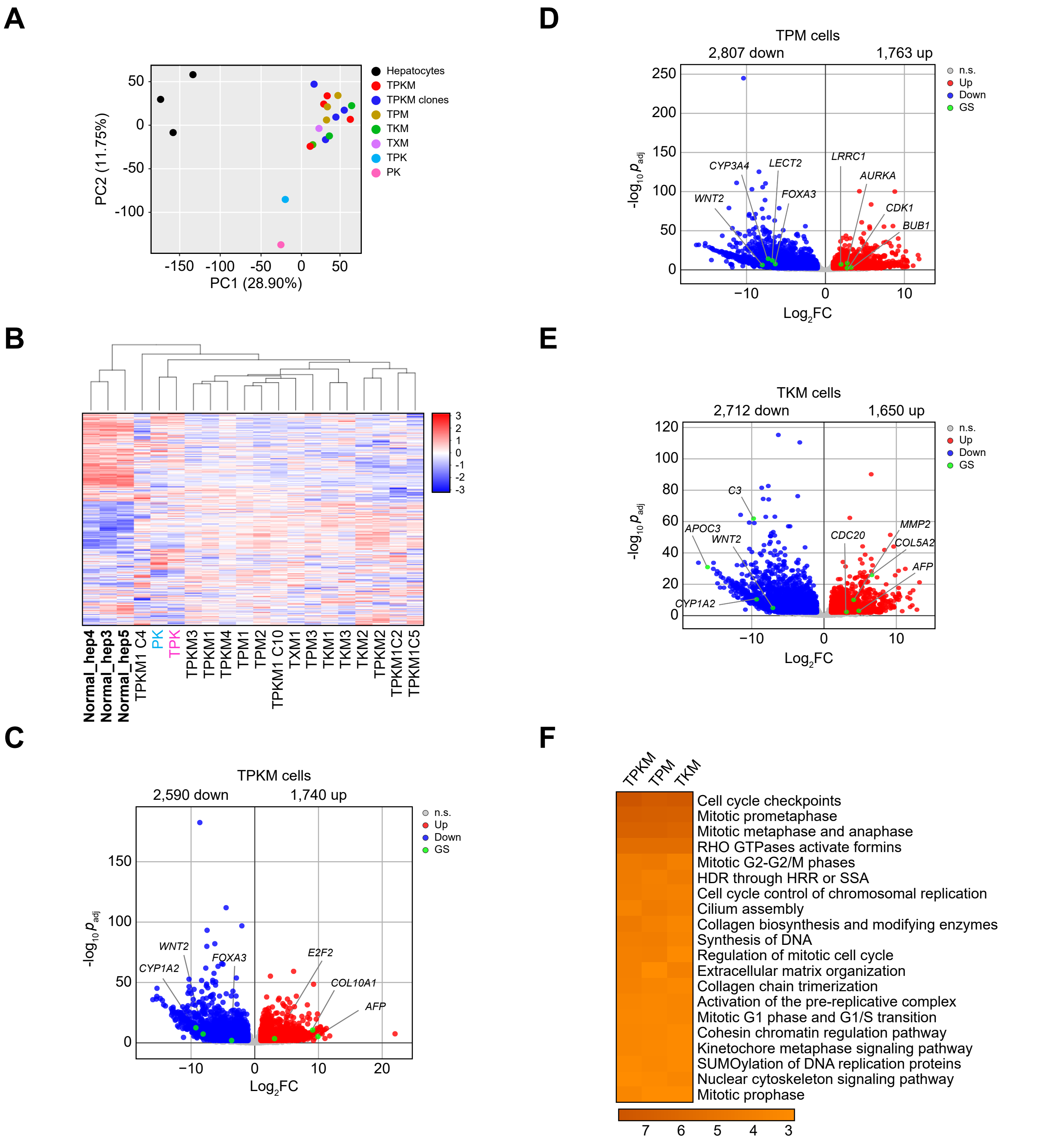


**Fig. 4. Transcriptional analysis of the established liver cancer cells reveals cell cycle activation and downregulation of liver function pathways.** (A) PC analysis of gene expression in unmodified control porcine hepatocytes derived from three independent pigs and established cells. (B) Unsupervised clustering heatmap of transcriptome-wide expression profiles. Numbers indicate the donor pig from which each cell line was derived. Z-score color scale shown. (C-E) Volcano plots of differential gene expression in TPKM (C), TPM (D), and TKM (E) cells *vs.* control hepatocytes ($q$ <0.05, |$\log_2$ fold change| >1). 10% of non-significant (n.s.) genes are shown in gray. Selected key genes of interest (GS) are labeled. (F) Top 20 activated pathways in TPKM, TPM, and TKM cells compared with control hepatocytes, ranked by z-scores. Z-score color scale shown. HDR, homology-directed repair; HRR, homologous recombination; PC, principal component; SSA, single strand annealing.

and unsupervised clustering revealed that tumors developed at gelatin sponge injection sites (masses 1 and 2) segregated distinctly from normal liver, while tumors developed at hydrogel injection sites (masses 3 and 4) clustered more closely with normal liver (Fig. 7A,B). Consistent with these findings, gelatin sponge tumors exhibited 5,748 DEGs

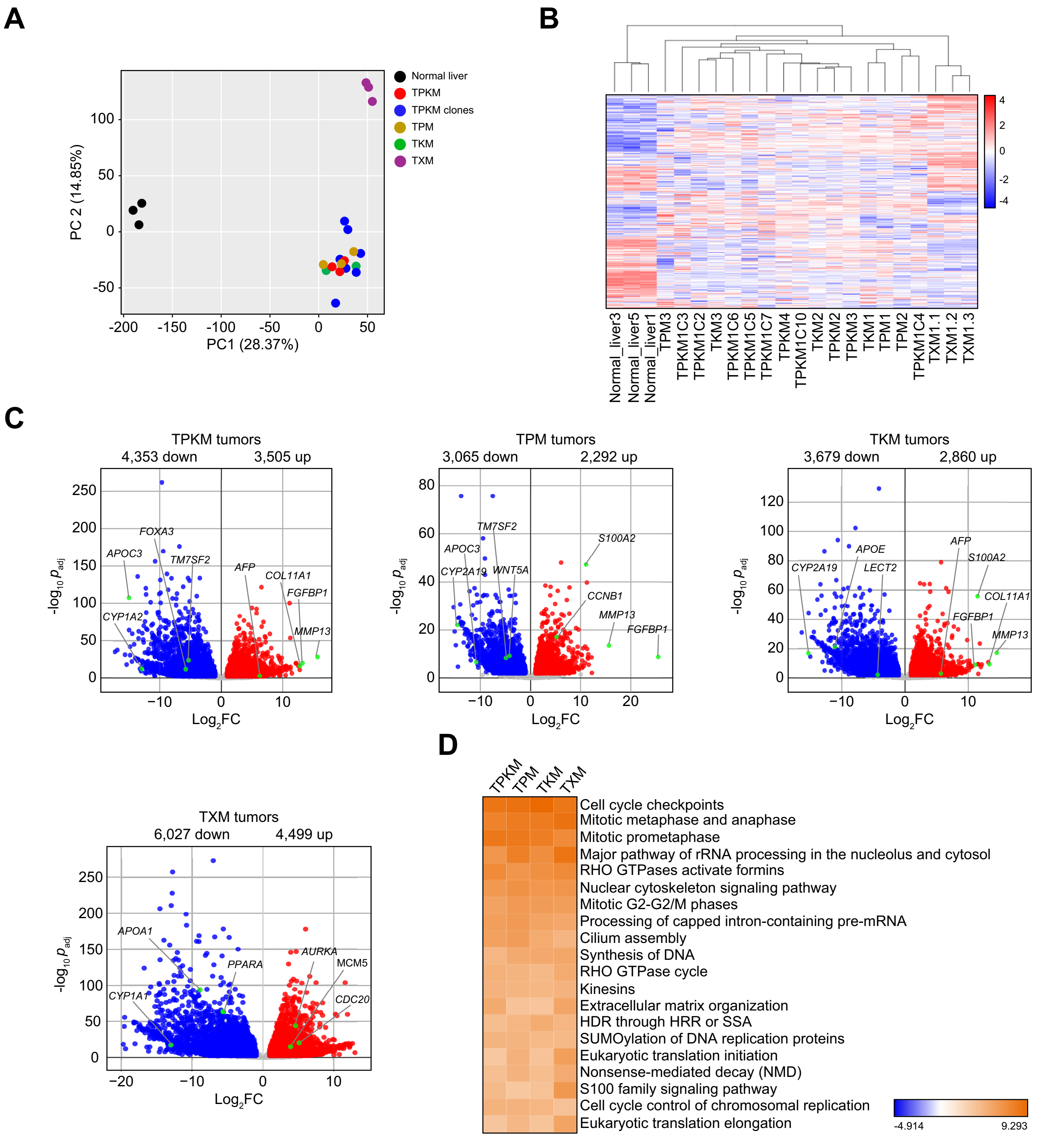


**Fig. 5. Transcriptional analysis of xenograft tumors developed from transformed porcine hepatocytes.** (A,B) PC analysis (A) and unsupervised clustering heatmap (B) of transcriptome-wide expression profiles of TPKM, TPM, TKM, and TXM xenograft tumors developed in SCID mice compared with normal pig liver. Numbers indicate the donor pig from which the corresponding cell line was derived. Z-score color scale shown. (C) Volcano plots showing differentially expressed genes in TPKM, TPM, TKM, and TXM xenograft tumors versus control liver ($q$ <0.05, $|\log_2$ fold change$| > 1$). 10% of non-significant genes are shown in gray. Selected key genes of interest are annotated. (D) Top 20 significantly enriched pathways in TPKM, TPM, TKM, and TXM xenograft tumors compared with control liver, ranked by z-score. Z-score color scale shown. HDR, homology-directed repair; HRR, homologous recombination; PC, principal component; SSA, single strand annealing.

A

| Pig no. | Injected cells & scaffold | No. of injected cells/site | No. of masses developed |
|---|---|---|---|
| 2 | TPKM, TPM, TKM, PK (hydrogel) | 15 million | None |
| 3 | TPKM* 4 sites (gelatin sponge/hydrogel) | 70 million | 4 masses |
| 4 | TPKM* 4 sites (gelatin sponge/hydrogel) | 55 million | 3 masses |
| 6 | TPKM* 4 sites (Matrigel/Vitrogel) | 30 Million | None |
| 7 | TPKM* 4 sites (Matrigel/Vitrogel) | 30 Million | None |

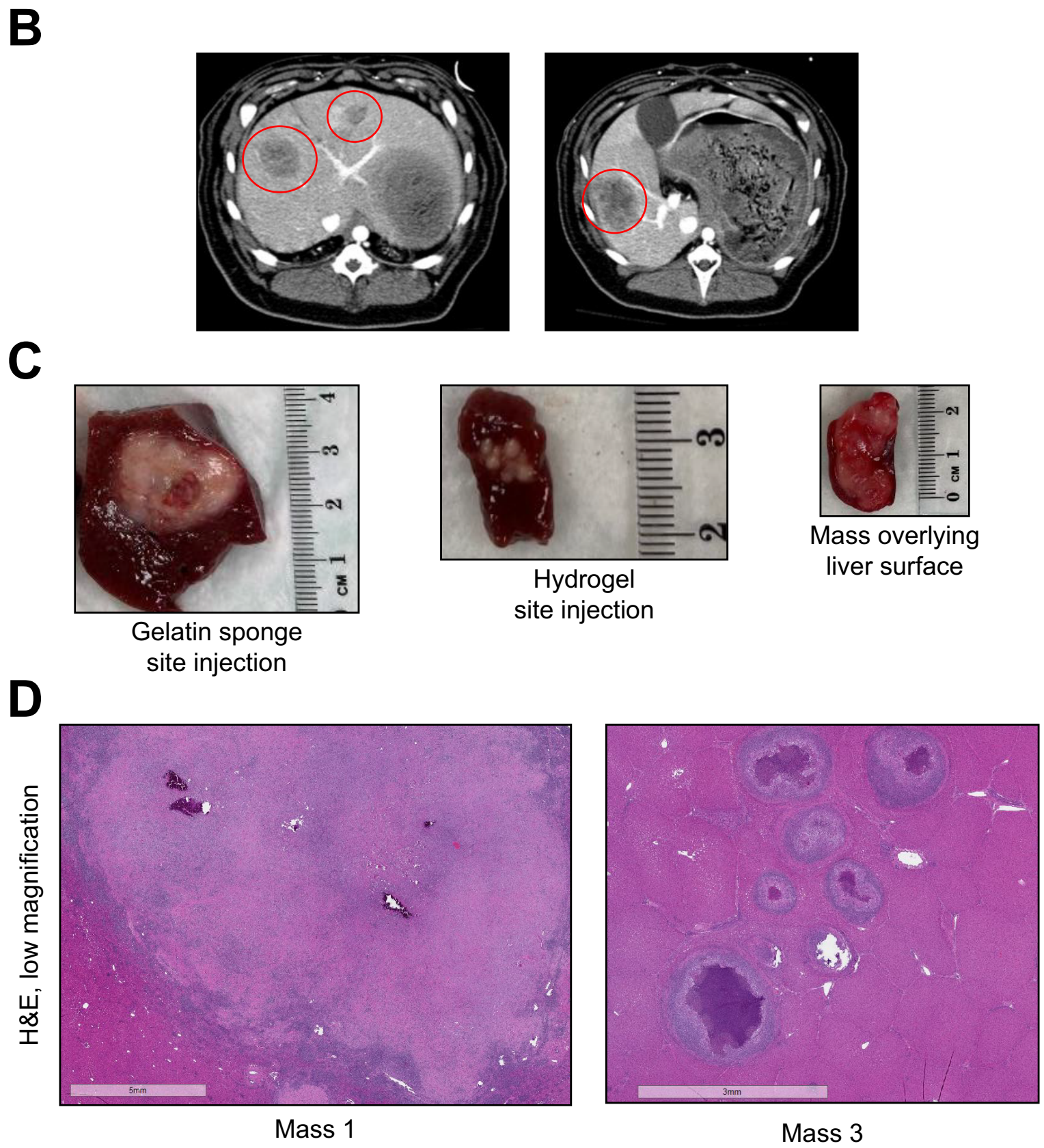


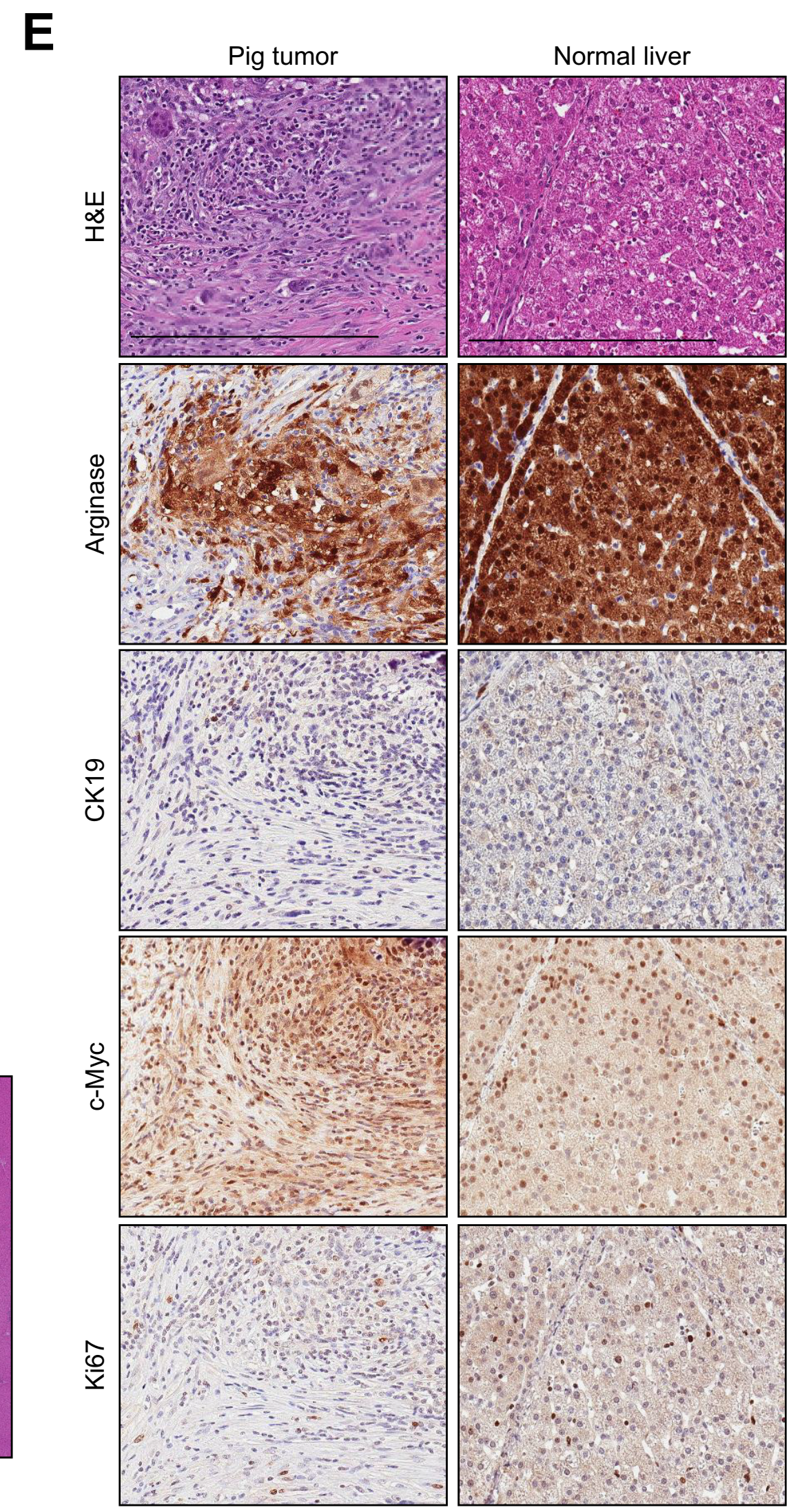


**Fig. 6. Development of liver tumors in pigs by orthotopic implantation of autologous transformed hepatocytes.** Cells developed by hepatocyte gene alteration were autologously injected into four liver sites per pig (n = 5 pigs) under ultrasound guidance. (A) Table summarizing injected cell lines, scaffold materials, number of injected cells per site, and tumor incidence in each pig. (B) Representative abdominal triple-phase contrast-enhanced CT scan from Pig 3 at 5 weeks post-injection, showing three liver masses (circled in red). (C) Representative images of gross tumors harvested from Pig 3 at 7 weeks post-injection at gelatin sponge (transected) or hydrogel injection sites, as well as a mass overlying the liver surface. A centimeter ruler is shown for scale. (D) Low-magnification H&E images of mass 1 from Pig 3, showing a large tumor mass at the gelatin sponge injection site (scale bar, 5 mm), and mass 3 from Pig 3, demonstrating small tumor nodules surrounded by normal liver tissue at the hydrogel injection site (scale bar, 3 mm). (E) Representative sections from mass 1 of Pig 3 stained with H&E, arginase, CK19, c-Myc, and Ki67. Scale bar, 300 μm. Higher-magnification H&E images of the same tumor section demonstrating tumor immune cell infiltration and calcification are shown in Fig. S8.

compared to normal liver, whereas hydrogel tumors showed only 453 DEGs (Fig. 7C,D; Table S8). The relatively small number of DEGs in masses 3 and 4, together with the considerable normal liver tissue surrounding these tumor nodules observed histologically (Fig. 6D), suggests that these bulk RNA-seq samples contained a higher proportion of normal liver tissue.

Pathway analysis of the tumors developed with both scaffolds revealed upregulation of cancer and cell proliferation pathways, including molecular mechanisms of cancer, ECM remodeling, Rho GTPase signaling, as well as focal adhesion kinase and integrin signaling (Fig. 7E, Table S9). Upregulated genes included *MMP2*, *MMP9*, *TGFB*, *FYN*, and multiple collagen and integrin genes. Masses developed at gelatin sponge injection sites also showed upregulation of *CCND2*, *CCN5*, *PIK3CD*, and *PAK1* (Table S8). Consistent with the immune cell infiltration observed histologically, pathway analysis revealed upregulation of multiple immune and inflammatory pathways, including the Th1 pathway, T cell receptor (TCR) signaling, phagosome formation, and leukocyte extravasation. Relevant upregulated genes included *CD28*, *CD3D*, *CD3E*, *ITK*, *NFATC2*, *GATA3*, *IL18R1*, and *JAK3*. Downregulated pathways in tumors at gelatin sponge injection sites were primarily associated with normal liver function, including lipid and cholesterol metabolism, xenobiotic and drug metabolism pathways, amino acid

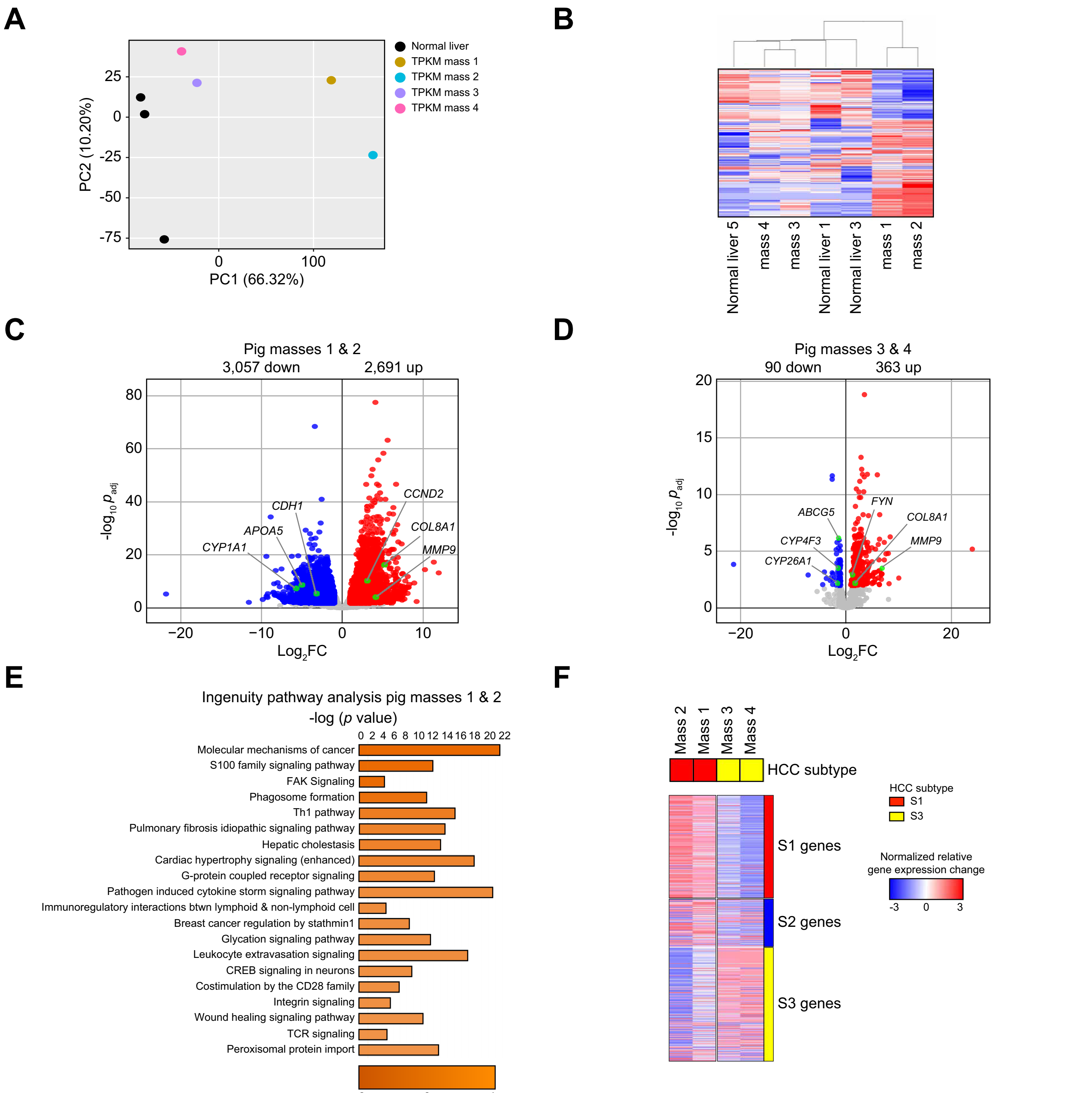


**Fig. 7. Transcriptional analysis reveals activation of cancer and immune pathways in the porcine intrahepatic tumors.** (A, B) PC analysis (A) and unsupervised clustering heatmap (B) of transcriptome-wide expression profiles of four porcine intrahepatic tumors harvested from Pig 3 and normal liver controls (pigs 2, 3, and 5). Masses 1 and 2 developed at injection sites using gelatin sponge as a scaffold, while masses 3 and 4 developed at injection sites using synthetic hydrogel. (C, D) Volcano plots showing differentially expressed genes in masses 1/2 (C) and masses 3/4 (D) *vs.* control liver ($q$ <0.05, $|\log_2$ fold change$|$ >1). 10% of non-significant genes are shown in gray. Selected key genes of interest are annotated. (E) Top 20 activated pathways in porcine masses 1/2 compared with control liver, ranked by z-score. Z-score color scale shown. (F) Expression heatmap of the Hoshida 619-gene HCC subtype signature in porcine masses 1-4. Z-score color scale shown. HCC, hepatocellular carcinoma; PC, principal component.

metabolism, and energy production. Downregulated genes included *APOA1*, *APOB*, *APOC3*, *ALB*, *CYP2E1*, *CYP1A2*, and *CYP1A1*. Tumors developed at gelatin sponge injection sites exhibited transcriptomic features consistent with the Hoshida S1 subtype (Fig. 7F), supported by their observed ECM remodeling and fibrosis. In contrast, tumors developed at hydrogel injection sites corresponded to the S3 subtype (Fig. 7F), which is characterized by normal hepatocyte

functional signatures. The substantial normal liver tissue surrounding the tumor nodules (Fig. 6C,D), and thus inevitably included in the bulk RNA-seq samples, likely contributed to their S3 classification.

To further characterize the tumor microenvironment, we analyzed bulk RNA sequencing data from masses 1 and 2 using MCP-counter to estimate immune and stromal cell populations.[24] Compared with normal liver tissue, tumors demonstrated increased abundance of multiple immune and stromal populations, including T cells, CD8+ T cells, cytotoxic lymphocytes, B-lineage cells, monocytic lineage cells, myeloid dendritic cells, endothelial cells, and fibroblasts (Fig. S9A). MCP-counter marker genes similarly showed increased expression of immune and stromal transcripts in tumors (Fig. S9B). Analysis of HCC immune class signature genes revealed enrichment of immune activation markers (*CD8A*, *GZMA*, *PRF1*), immune checkpoint-associated genes (*PDCD1*, *CTLA4*, *LAG3*), and stromal/TGF-β-associated genes (*TGFB1*, *COL1A1*, *POSTN*) (Fig. S9C). These findings indicate an immune-infiltrated tumor microenvironment with features associated with immune exhaustion and stromal activation.[25]

Together, these findings provide proof of principle for the development of orthotopic HCC implantation models in immune-competent, wild type pigs, where tumors can be engineered to harbor defined gene alterations.

## Discussion

Improving outcomes for patients with liver cancer requires robust preclinical models to evaluate emerging therapeutic approaches. Here, we developed liver cancer cell and animal models through CRISPR editing of primary hepatocytes. Introducing distinct gene alterations into porcine hepatocytes revealed combinations capable of driving hepatocyte transformation *ex vivo* and yielded genetically defined liver cancer cell models. These cells generated tumors in immunodeficient mice that recapitulated key histological and molecular features of human liver cancer, and autologous orthotopic implantation into pigs established proof of concept for an immune-competent large animal model.

Large-scale sequencing studies have shown that HCC tumors harbor 35 to 80 coding mutations per tumor, including 4 to 8 predicted driver mutations.[35] However, as clinical tumors are detected only after substantial progression, sequencing cannot determine which alterations were responsible for HCC initiation. By applying CRISPR editing directly to primary adult hepatocytes, our study provides a more physiologically relevant system compared with models that use fibroblast-induced hepatocytes. We found that knockout of only two tumor suppressor genes (TP, TK, or PK) was insufficient for *ex vivo* hepatocyte transformation, whereas at least three alterations were required. This aligns with observations in human cells, which typically require multiple genetic hits, and contrasts with murine cells, where two alterations often suffice.[10] The higher transformation threshold of porcine hepatocytes, consistent with findings in porcine fibroblasts,[36] underscores the value of pigs as a stringent and translationally relevant model system.

Inducing gene alterations in hepatocytes enabled the development of genetically tailored liver cancer cells that exhibit activation of cell cycle and ECM remodeling pathways and express gene signatures frequently observed in human HCC. A key strength of this strategy is the ability to generate isogenic cancer cell models on a normal genetic background, avoiding the numerous confounding mutations present in HCC cell lines derived from patient tumors. Hence, these cell models provide a tractable platform suitable for assessing the impact of gene alterations on HCC initiation, progression, and therapeutic susceptibility.

The translational relevance of our established cell models was further underscored by their *in vivo* histological and transcriptional features. TPKM, TPM, and TKM xenograft tumors contained both HCC and CCA areas. This is consistent with a recent study showing that sgTP53+sgPTEN generated combined HCC-CCA tumors in mice.[37] The presence of CCA areas likely reflects HCC-to-CCA transdifferentiation, which was recently shown to be promoted by c-Myc.[38] TXM tumors, by contrast, resembled HCC histologically without a CCA component, which could be attributed to the lower c-Myc expression levels in TXM cells. Our study also highlights an important role for *c-Myc* in porcine hepatocarcinogenesis. While TP and TK cells failed to form tumors *in vivo*, addition of c-Myc overexpression (TPM and TKM) resulted in tumor formation, indicating that c-Myc cooperates with tumor suppressor gene alterations to promote malignant transformation. Further supporting a role for c-Myc in porcine HCC pathogenesis, tumors arising from cells with *TP53*, *PTEN*, and *CDKN2A* alterations alone (TPK) did not resemble HCC, whereas otherwise similar cells with *c-Myc* overexpression (TPKM) yielded tumors with phenotypic features consistent with liver cancer. These findings are consistent with observations in both human HCC and genetically engineered mouse models, where MYC plays an important role in hepatocarcinogenesis.[26,39–42] Importantly, TPKM, TPM, TKM, and TXM xenograft tumors exhibited transcriptional features similar to those observed in human HCC. Collectively, these findings delineate oncogenic cooperations underlying HCC pathogenesis and establish a versatile system that can be used to dissect driver gene cooperation.

Large-animal models are essential for advancing LRTs, which can be tailored to tumor mutations, particularly as driver mutations have been shown to influence HCC response to LRTs in clinical studies.[43,44] Through autologous intrahepatic implantation of transformed hepatocytes, we generated genetically defined tumors in immune-competent pigs. This approach circumvents the cost and long timelines of generating transgenic pigs, while allowing the introduction of tailored gene alterations. Compared with the widely used rabbit VX2 model, which represents a squamous carcinoma,[45] and chemically induced porcine tumors, which do not allow precise genetic manipulation of tumors,[46] this model provides greater biological and translational relevance.

A limitation of our study is that CRISPR-mediated editing produced heterogeneous indels, including in-frame variants that may not result in complete loss-of-function, thereby complicating interpretation of gene-specific effects. Further, protein-level validation of tumor suppressor gene disruption was not performed owing to the limited availability of antibodies with demonstrated cross-reactivity to porcine proteins.[47] In addition, several genotypes were evaluated in a limited number of mice, and only a limited number of clonal lines were analyzed, which may explain why certain histological features observed in pooled-cell tumors were absent in

clonal derivatives. Another practical limitation of the porcine model is the need to generate pig-specific cell lines for autologous injections, a time- and labor-intensive step that contributed to the small cohort size in the study, in addition to the inherent sample size constraints of large-animal studies.[47] Additionally, using only male pigs without a cirrhotic background precluded assessment of sex-specific effects and the role of underlying liver disease.

Future studies incorporating cirrhosis, expanding pig cohorts, including both sexes, performing more comprehensive immune profiling, and validating protein-level consequences of gene disruption and downstream pathway alterations will further strengthen the model's utility for translational research. In addition, development of models harboring alternative driver alterations will further expand the molecular and histological diversity represented by this platform. Given the prevalence of activating *CTNNB1* mutations in human HCC,[3–5,22,27] generation of β-catenin-driven porcine HCC models using CRISPR-based approaches similar to those employed in mouse models[48,49] represents a valuable future direction.

In summary, we established novel cell, xenograft, and porcine models of liver cancer that recapitulate key features of human HCC through combinatorial gene alterations frequently observed in patient tumors. This modeling platform holds promise for accelerating the development of effective, precision therapies for HCC, including both locoregional and systemic therapies.

## Affiliations

[1]*Department of Radiology, University of Illinois at Chicago, Chicago, Illinois, USA;* [2]*Division of Digestive and Liver Diseases, Department of Internal Medicine, University of Texas Southwestern Medical Center, Dallas, USA;* [3]*Department of Veterinary Clinical Medicine, University of Illinois at Urbana-Champaign, Champaign, Illinois, USA;* [4]*Department of Oncological Sciences, Icahn School of Medicine at Mount Sinai, New York, New York, USA;* [5]*Department of Animal Sciences, University of Illinois at Urbana-Champaign, Urbana, Illinois, USA;* [6]*Department of Pathology, University of Illinois at Chicago, Chicago, Illinois, USA*

## Abbreviations

AFP, alpha-fetoprotein; CCA, cholangiocarcinoma; DEG, differentially expressed gene; ECM, extracellular matrix; HCC, hepatocellular carcinoma; LRT, locoregional therapy; OE, overexpression; PCA, principal component analysis; RNA-seq, RNA sequencing; RNP, ribonucleoprotein; SCID, severe combined immunodeficiency; sgRNA, single-guide RNA.

## Financial support

This work was supported by a Society of Interventional Radiology (SIR) Foundation grant to L.E., an American Cancer Society Institutional Research grant (ACS-IRG) to L.E., and funding from the Department of Radiology, University of Illinois Chicago. L.E. is supported by NIH R21CA292350. Y.H. is supported by NIH (R01CA233794, R01CA255621, R01CA282178, R01CA292930, U01CA288375, U01CA283935, P50CA295495); European Commission (ERC-AdG-2020-101021417); and Cancer Prevention and Research Institute of Texas (RR180016, RP200554). R.C.G. is supported by R01CA283548.

## Conflicts of interest

L.R.J. reports working for Sus Clinicals. Y.H. is an advisor for Helio Genomics, Espervita Therapeutics, Roche Diagnostics; shareholder for Alentis Therapeutics, Espervita Therapeutics. L.B.S. and K.M.S. report grants from Janssen Research & Development LLC and Earli Inc.; and work for Sus Clinicals. R.C.G. reports grants from Guerbet USA LLC, Sus Clinicals, Inc.; consulting fees from ClearDynamic; participation on Sus Clinicals, Inc. Scientific Advisory Board, Fluidx Medical Technology Advisory board, and Kaveri University Advisory board; and stock or stock options in Sus Clinicals, Inc. The remaining authors declare no conflicts of interest.

Please refer to the accompanying ICMJE disclosure forms for further details.

## Authors' contributions

Conceptualization, L.E., K.M.S., R.C.G.; Methodology, L.E., L.B.S., A.L., K.M.S, R.C.G.; Formal analysis, L.E., O.G.D.; Investigation, L.E., O.G.D., C.C.C., L.N.R., L.R.J., N.I.K., H.L., I.A., Y.Z., H.K., Y.H., J.P.S., G.G., R.C.G.; Writing - Original Draft, L.E.; Writing - Review & Editing, R.C.G., K.M.S.; Visualization, L.E., O.G.D., H.K.; Project administration, L.E.; Funding acquisition, L.E.

## Data and materials availability

The raw and processed RNA-seq data generated in this study have been deposited in the NCBI Gene Expression Omnibus (GEO) under accession number GSE311743. The cell lines generated in this study are available from the corresponding author upon reasonable request.

## Acknowledgments

We thank the veterinarians and staff at the University of Illinois Chicago (UIC) animal facility for support with pig surgery and husbandry, the UIC Research Histology Core, the UIC Research Tissue Imaging Core, the UIC Genome Research Core, the University of Illinois Urbana-Champaign DNA Services Laboratory, and the UIC Center for Clinical and Translational Science (CCTS) Biostatistics Core for technical support. We are also grateful to Eleanor Li for assistance with immunohistochemistry optimization and to Vishwaarth Vijayakumar for help with RNA-seq analysis.

## Supplementary data

Supplementary data to this article can be found online at https://doi.org/10.1016/j.jhepr.2026.102042.

## References

*Author names in bold designate shared co-first authorship*

[1] Sung H, Ferlay J, Siegel RL, et al. Global cancer Statistics 2020: GLOBOCAN estimates of incidence and mortality worldwide for 36 cancers in 185 Countries. CA Cancer J Clin 2021;71:209–249.
[2] Llovet JM, Kelley RK, Villanueva A, et al. Hepatocellular carcinoma. Nat Rev Dis Primers 2021;7:6.
[3] Cancer Genome Atlas Research Network. Electronic address wbe, cancer genome atlas Research N. Comprehensive and integrative genomic characterization of hepatocellular carcinoma. Cell 2017;169:1327–1341 e1323.
[4] **Fujimoto A**, **Furuta M**, **Totoki Y**, et al. Whole-genome mutational landscape and characterization of noncoding and structural mutations in liver cancer. Nat Genet 2016;48:500–509.
[5] **Schulze K**, **Imbeaud S**, **Letouze E**, et al. Exome sequencing of hepatocellular carcinomas identifies new mutational signatures and potential therapeutic targets. Nat Genet 2015;47:505–511.
[6] Holczbauer A, Factor VM, Andersen JB, et al. Modeling pathogenesis of primary liver cancer in lineage-specific mouse cell types. Gastroenterology 2013;145:221–231.
[7] Pez F, Gifu P, Degli-Esposti D, et al. In vitro transformation of primary human hepatocytes: epigenetic changes and stemness properties. Exp Cel Res 2019;384:111643.
[8] Sun L, Wang Y, Cen J, et al. Modelling liver cancer initiation with organoids derived from directly reprogrammed human hepatocytes. Nat Cel Biol 2019;21:1015–1026.
[9] Xie S, Zhu M, Lv G, et al. Overexpression of Ras homologous C (RhoC) induces malignant transformation of hepatocytes in vitro and in nude mouse xenografts. PLoS One 2013;8:e54493.
[10] Rangarajan A, Weinberg RA. Opinion: Comparative biology of mouse versus human cells: modelling human cancer in mice. Nat Rev Cancer 2003;3:952–959.
[11] Singh M, Murriel CL, Johnson L. Genetically engineered mouse models: closing the gap between preclinical data and trial outcomes. Cancer Res 2012;72:2695–2700.
[12] Aravalli RN, Golzarian J, Cressman EN. Animal models of cancer in interventional radiology. Eur Radiol 2009;19:1049–1053.

[13] Greten TF, Mauda-Havakuk M, Heinrich B, et al. Combined locoregional-immunotherapy for liver cancer. J Hepatol 2019;70:999–1007.
[14] Llovet JM, De Baere T, Kulik L, et al. Locoregional therapies in the era of molecular and immune treatments for hepatocellular carcinoma. Nat Rev Gastroenterol Hepatol 2021;18:293–313.
[15] Hryhorowicz M, Lipinski D, Hryhorowicz S, et al. Application of genetically engineered pigs in Biomedical Research. Genes (Basel) 2020;11.
[16] Gaba RC, Elkhadragy L, Boas FE, et al. Development and comprehensive characterization of porcine hepatocellular carcinoma for translational liver cancer investigation. Oncotarget 2020;11:2686–2701.
[17] Jaroch DB, Liu Y, Kim AY, et al. Pressure-enabled drug Delivery significantly Increases Intra-arterial Delivery of embolic Microspheres to liver tumors in a porcine model. J Vasc Interv Radiol 2025;36:499–504 e491.
[18] Nurili F, Monette S, Michel AO, et al. Transarterial embolization of liver cancer in a transgenic pig model. J Vasc Interv Radiol 2021;32:510–517 e513.
[19] Elkhadragy L, Regan MR, Totura WM, et al. Generation of genetically tailored porcine liver cancer cells by CRISPR/Cas9 editing. Biotechniques 2021;70:37–48.
[20] Elkhadragy L, Carlino MJ, Jordan LR, et al. Development of a genetically tailored implantation hepatocellular carcinoma model in Oncopigs by somatic cell CRISPR editing. Dis Model Mech 2025;18.
[21] Elkhadragy L, Dasteh Goli K, Totura WM, et al. Effect of CRISPR knockout of AXIN1 or ARID1A on proliferation and Migration of porcine hepatocellular carcinoma. Front Oncol 2022;12:904031.
[22] Hoshida Y, Nijman SM, Kobayashi M, et al. Integrative transcriptome analysis reveals common molecular subclasses of human hepatocellular carcinoma. Cancer Res 2009;69:7385–7392.
[23] Dhanasekaran R, Suzuki H, Lemaitre L, et al. Molecular and immune landscape of hepatocellular carcinoma to guide therapeutic decision-making. Hepatology 2025;81:1038–1057.
[24] Becht E, Giraldo NA, Lacroix L, et al. Estimating the population abundance of tissue-infiltrating immune and stromal cell populations using gene expression. Genome Biol 2016;17:218.
[25] Sia D, Jiao Y, Martinez-Quetglas I, et al. Identification of an immune-specific class of hepatocellular carcinoma, based on molecular features. Gastroenterology 2017;153:812–826.
[26] Molina-Sanchez P, Ruiz de Galarreta M, Yao MA, et al. Cooperation between distinct cancer driver genes underlies inter-tumor heterogeneity in hepatocellular carcinoma. Gastroenterology 2020;159:2203–2220.e14.
[27] Rebouissou S, Nault JC. Advances in molecular classification and precision oncology in hepatocellular carcinoma. J Hepatol 2020;72:215–229.
[28] Wang G, Chow RD, Ye L, et al. Mapping a functional cancer genome atlas of tumor suppressors in mouse liver using AAV-CRISPR-mediated direct in vivo screening. Sci Adv 2018;4:eaao5508.
[29] Zender L, Xue W, Zuber J, et al. An oncogenomics-based in vivo RNAi screen identifies tumor suppressors in liver cancer. Cell 2008;135:852–864.
[30] **Kaur I**, **Vasudevan A**, Rawal P, et al. Primary hepatocyte isolation and cultures: technical Aspects, Challenges and advancements. Bioengineering (Basel) 2023;10.
[31] Zhuo JY, Lu D, Tan WY, et al. CK19-positive hepatocellular carcinoma is a characteristic subtype. J Cancer 2020;11:5069–5077.
[32] Hua S, Ji Z, Quan Y, et al. Identification of hub genes in hepatocellular carcinoma using integrated bioinformatic analysis. Aging (Albany NY) 2020;12:5439–5468.
[33] Jiang N, Zhang X, Qin D, et al. Identification of Core genes related to progression and prognosis of hepatocellular carcinoma and small-Molecule drug Predication. Front Genet 2021;12:608017.
[34] Schlegel P, Yan K, Upadhyaya S, et al. Tissue-engineered vocal fold replacement in swine: Methods for functional and structural analysis. PLoS One 2023;18:e0284135.
[35] Zucman-Rossi J, Villanueva A, Nault JC, et al. Genetic landscape and biomarkers of hepatocellular carcinoma. Gastroenterology 2015;149:1226–1239 e1224.
[36] Adam SJ, Rund LA, Kuzmuk KN, et al. Genetic induction of tumorigenesis in swine. Oncogene 2007;26:1038–1045.
[37] Tang M, Zhao Y, Zhao J, et al. Liver cancer heterogeneity modeled by in situ genome editing of hepatocytes. Sci Adv 2022;8:eabn5683.
[38] D'Artista L, Moschopoulou AA, Barozzi I, et al. MYC determines lineage commitment in KRAS-driven primary liver cancer development. J Hepatol 2023;79:141–149.
[39] Dai P, Wang L. Targeting c-MYC has a key role in hepatocellular carcinoma therapy. Crit Rev Oncol Hematol 2025;213:104786.
[40] Kaposi-Novak P, Libbrecht L, Woo HG, et al. Central role of c-Myc during malignant conversion in human hepatocarcinogenesis. Cancer Res 2009;69:2775–2782.
[41] Qu A, Jiang C, Cai Y, et al. Role of Myc in hepatocellular proliferation and hepatocarcinogenesis. J Hepatol 2014;60:331–338.
[42] Zheng K, Cubero FJ, Nevzorova YA. c-MYC-Making liver Sick: role of c-MYC in hepatic cell function, Homeostasis and disease. Genes (Basel) 2017;8.
[43] Weinfurtner K, Crainic JA, Tischfield D, et al. Distinct metabolic phenotype renders beta-catenin mutant hepatocellular carcinoma susceptible to treatment-induced ischemia. medRxiv 2024. https://doi.org/10.1101/2023.09.28.23296283.
[44] Zhao K, Karimi A, Kelly L, et al. TP53 mutation Predicts Worse survival and Earlier local progression in patients with hepatocellular carcinoma treated with Transarterial embolization. Curr Oncol 2025;32.
[45] Pascale F, Pelage JP, Wassef M, et al. Rabbit VX2 liver tumor model: a Review of clinical, biology, Histology, and tumor microenvironment characteristics. Front Oncol 2022;12:871829.
[46] Mitchell J, Tinkey PT, Avritscher R, et al. Validation of a preclinical model of Diethylnitrosamine-induced hepatic Neoplasia in Yucatan Miniature pigs. Oncology 2016;91:90–100.
[47] Elkhadragy L, Gaba RC, Niemeyer MM, et al. Translational relevance and future integration of the Oncopig cancer model in preclinical Applications. Annu Rev Anim Biosci 2025;13:465–481.
[48] Loesch R, Caruso S, Paradis V, et al. Deleting the beta-catenin degradation domain in mouse hepatocytes drives hepatocellular carcinoma or hepatoblastoma-like tumor growth. J Hepatol 2022;77:424–435.
[49] Mou H, Eskiocak O, Ozler KA, et al. CRISPR-induced exon skipping of beta-catenin reveals tumorigenic mutants driving distinct subtypes of liver cancer. J Pathol 2023;259:415–427.


Keywords: Hepatocellular carcinoma; oncogenic cooperation; hepatocyte transformation; HCC initiation; CRISPR/Cas9 gene editing; HCC models; large-animal model.

*Received 23 December 2025; received in revised form 11 September 2026; accepted 17 September 2026; Available online 23 September 2026*

Journal of Hepatology, Volume *8*

## Supplemental information

# Modeling liver cancer molecular and histological features by CRISPR editing of porcine hepatocytes

**Lobna Elkhadragy, Olayinka G. David, Caitlyn C. Castillo, Luke N. Redlon, Luke R. Jordan, Nusrat I. Khan, Hannah Liu, Isadora Andre Rosa Lopes, Yanqiong Zhou, Hiroaki Kanzaki, Yujin Hoshida, Jonathan P. Samuelson, Amaia Lujambio, Lawrence B. Schook, Grace Guzman, Kyle M. Schachtschneider, and Ron C. Gaba**

# Modeling liver cancer molecular and histological features by CRISPR editing of porcine hepatocytes

Lobna Elkhadragy, Olayinka G. David, Caitlyn C. Castillo, Luke N. Redlon, Luke R. Jordan, Nusrat Khan, Hannah Liu, Isadora Andre Rosa Lopes, Yanqiong Zhou, Hiroaki Kanzaki, Yujin Hoshida, Jonathan P. Samuelson, Amaia Lujambio, Lawrence B. Schook, Grace Guzman, Kyle M. Schachtschneider, Ron C. Gaba

Table of contents

Supplementary Materials and Methods……………………………………………………2

Supplementary Figures……………………………………….……………………………..4

Supplementary Tables………………………………………...……...separate excel file

**Supplementary Materials and Methods**

**Western blotting:** Cells were washed with ice-cold PBS and lysed with RIPA buffer (Thermo Fisher #AAJ63306AK) containing protease inhibitor cocktail (Thermo Fisher #78429). Protein lysates were resolved by SDS–PAGE on 4-20% gels, transferred to nitrocellulose membranes, and blocked with 5% non-fat milk in PBS with tween 20 for 30 minutes. Membranes were incubated with primary antibodies overnight at 4°C, followed by 1 hour incubation with HRP-conjugated secondary antibodies at room temperature. The following antibodies were used: anti-c-Myc (Santa Cruz #9E10, sc-40), anti-PTEN (Santa Cruz sc-7974), anti-β-actin (Santa Cruz sc-47778), and anti-mouse secondary antibody (Biorad #170-6516). The Western blots were visualized by chemiluminescence (Thermo Fisher #32109).

**Doubling time analysis:** In vitro doubling time was determined by counting live cells that did not uptake Trypan blue dye at two time points and using the equation: doubling time = T ln2/ln(X2/X1), where T is the incubation time, X1 is the initial cell number, and X2 is the final cell number. Results were expressed as mean ± standard deviation (SD). Statistical comparisons were performed by one-way analysis of variance (ANOVA) using GraphPad Prism 10, with $p < 0.05$ considered statistically significant.

**Isolation of single-cell clones:** Cells were diluted to 1 cell/100 µl medium and seeded into 96-well plates. Wells were inspected microscopically to confirm the presence of a single cell/colony per well. Colonies reaching ~80-90% confluence were split into two wells: one for genomic DNA extraction and indel analysis, and the other for expansion.

**Histology and immunohistochemistry (IHC)**: Formalin-fixed, paraffin-embedded tissues were sectioned and stained with hematoxylin and eosin (H&E) and subjected to

IHC using antibodies against Arginase (Abcam #ab91279), CK19 (DSHB, Troma-III), c-Myc (Biocare Medical #PME415AA), and Ki67 (Agilent # M7240, MIB-1 clone). Whole slides were scanned at 20x magnification using the Leica Aperio AT2 slide scanner and digital images were visualized with Aperio ImageScope software (Leica).

**Ki67 quantification**: Digitized slides were analyzed in Halo (Indica Labs, v4.0.5107.488). Tissue segmentation into epithelium and stroma was performed using an AI-based mininet classifier, with three classifier sets required for segmenting all tissue in the analysis. Ki67 positivity was quantified in the tumor compartment using the Multiplex IHC v3.4.9 algorithm, with thresholds manually set and applied consistently across all samples. Tissue microarrays containing one or two representative tumor cores per tumor were analyzed. Data is expressed as the percentage of Ki67-positive tumor cells in tumor sections.

## Supplementary Figures

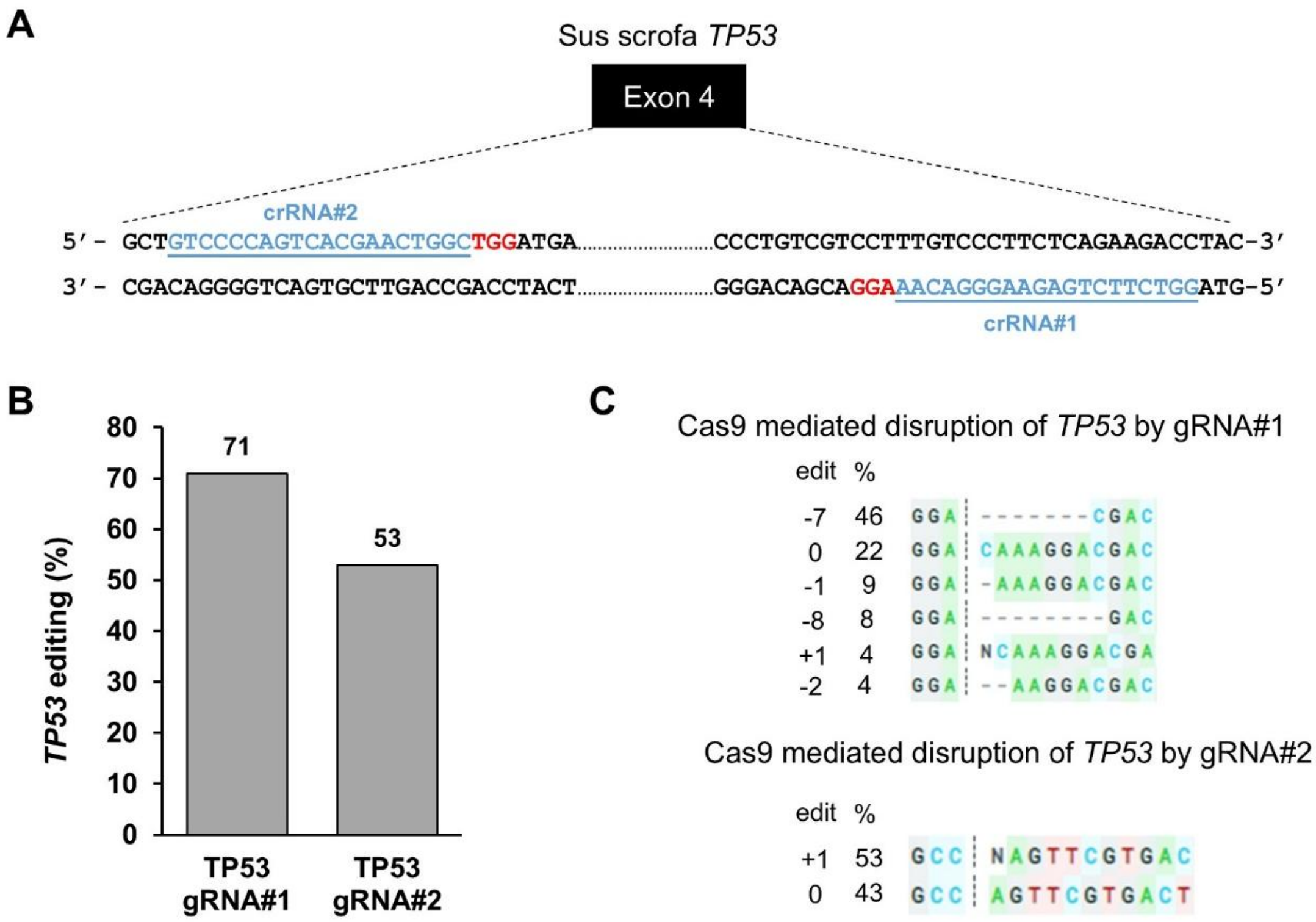


**Fig. S1. Screening CRISPR/Cas9 sgRNAs targeting porcine *TP53* gene.**

A) Schematic of the porcine *TP53* locus showing CRISPR spacer sequences crRNA#1 and crRNA#2 (blue, underlined). Protospacer adjacent motif (PAM) sequences are marked in red. B) Comparison of editing efficiency between the two *TP53*-targeting sgRNAs. Porcine cells (Oncopig A343 HCC cells, ref. #21) were transfected with each Cas9-sgRNA ribonucleoprotein complex and genomic DNA was extracted two days post-transfection for Sanger sequencing. Non-transfected cells were used as control. The bar graph shows the percentage (%) of reads with indels at each sgRNA target site, reflecting non-homologous end joining (NHEJ). C) Type and frequency of *TP53* indels detected by sequencing analysis. The percentage of reads for each sequence are shown on the left. Dashed line, predicted Cas9 cleavage position; N, nucleotide insertion; dash, deleted base.

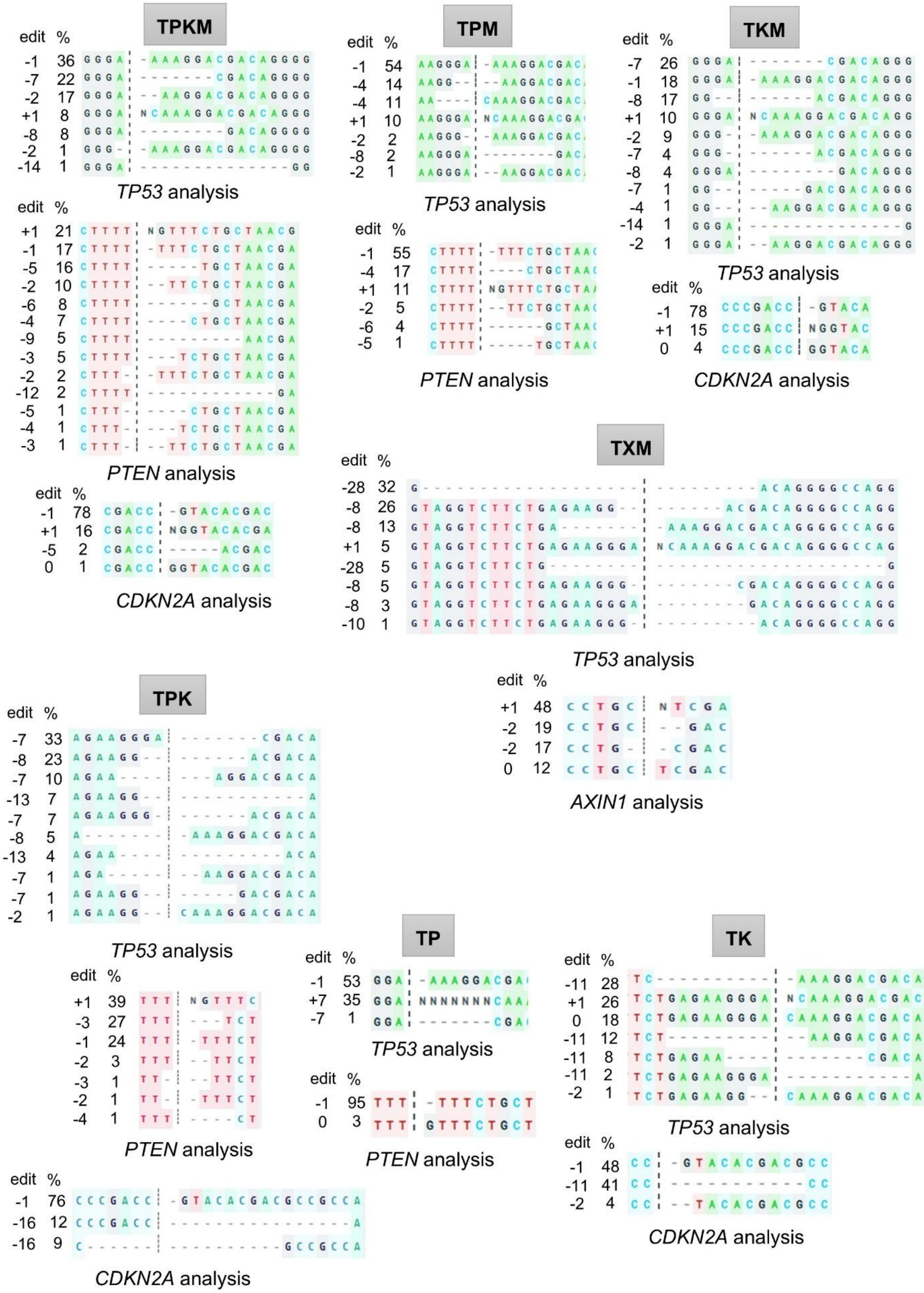


**Fig. S2. Validation of CRISPR gene editing in developed cell lines by sequencing analysis.**

Sequencing analysis confirmed CRISPR-induced edits in the targeted genes in the established cell lines. Representative indel analysis is shown for the following cells at the indicated passage (p) number, with pig of origin indicated: PG1-TPKM (p.8), PG1-TPM (p.9), PG1-TKM (p.5), PG2-TPK (p.17), PG1-TXM (p.17), PG3-TP (p.11), and PG3-TK (p.23). The type and frequency of indels are shown on the left. Dashed line, predicted Cas9 cleavage position; N, nucleotide insertion; dash, deleted base.

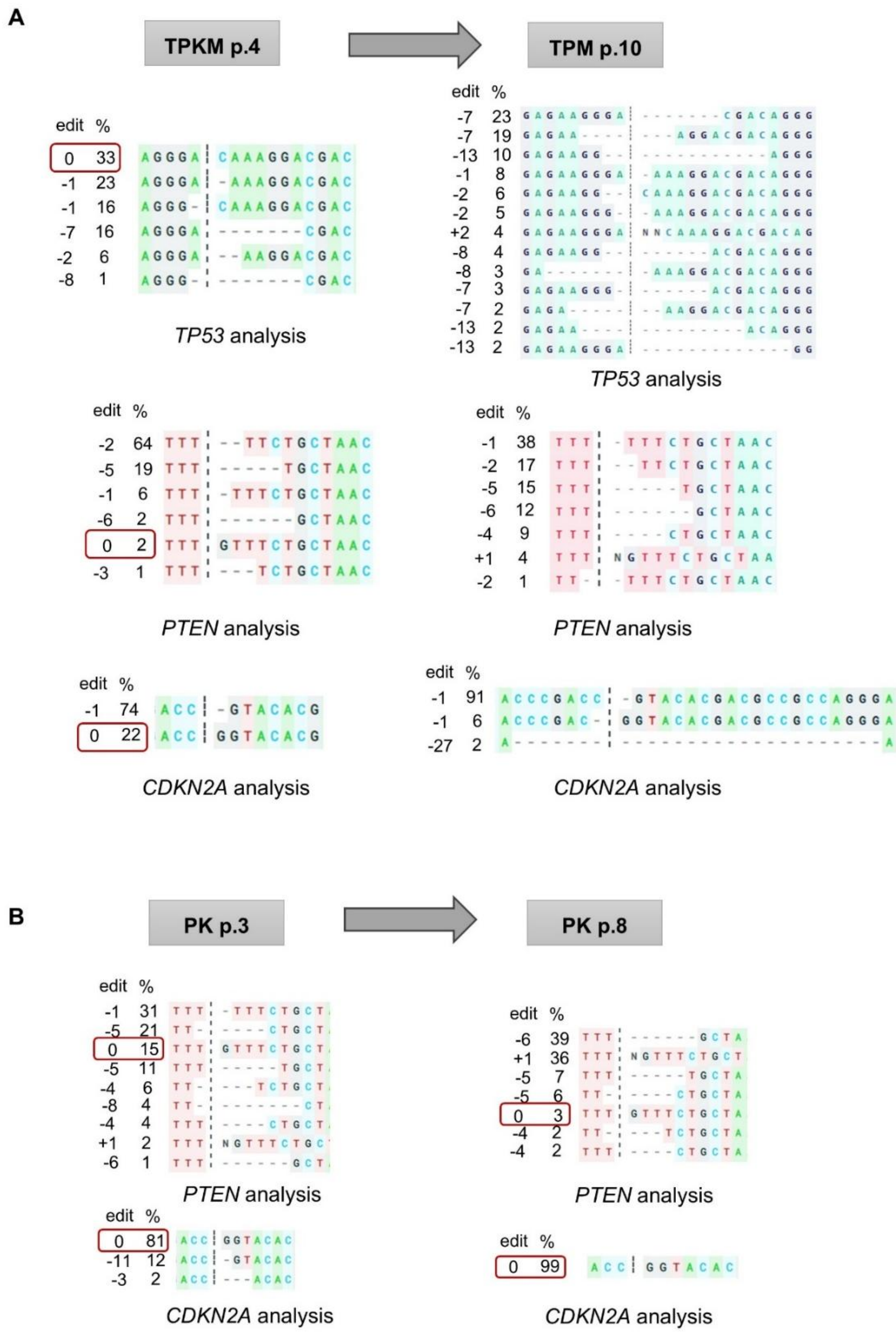


**Fig. S3. Variation of CRISPR-induced editing rates over successive passages in culture.**

A) Most cell lines showed enrichment of indels and depletion of unedited sequences in targeted tumor suppressor genes across cell passages. Representative sequencing of PG2-TPKM cells is shown, wherein unedited *TP53*, *PTEN*, and *CDKN2A* sequences were detectable at passage 4 but undetectable by passage 10, at which point all the three targeted genes contained indels. B) The only exception was the PK cell line,

which did not retain the induced indels in *CDKN2A* gene during culture, with all edited *CDKN2A* sequences being lost at later passages.

**A**

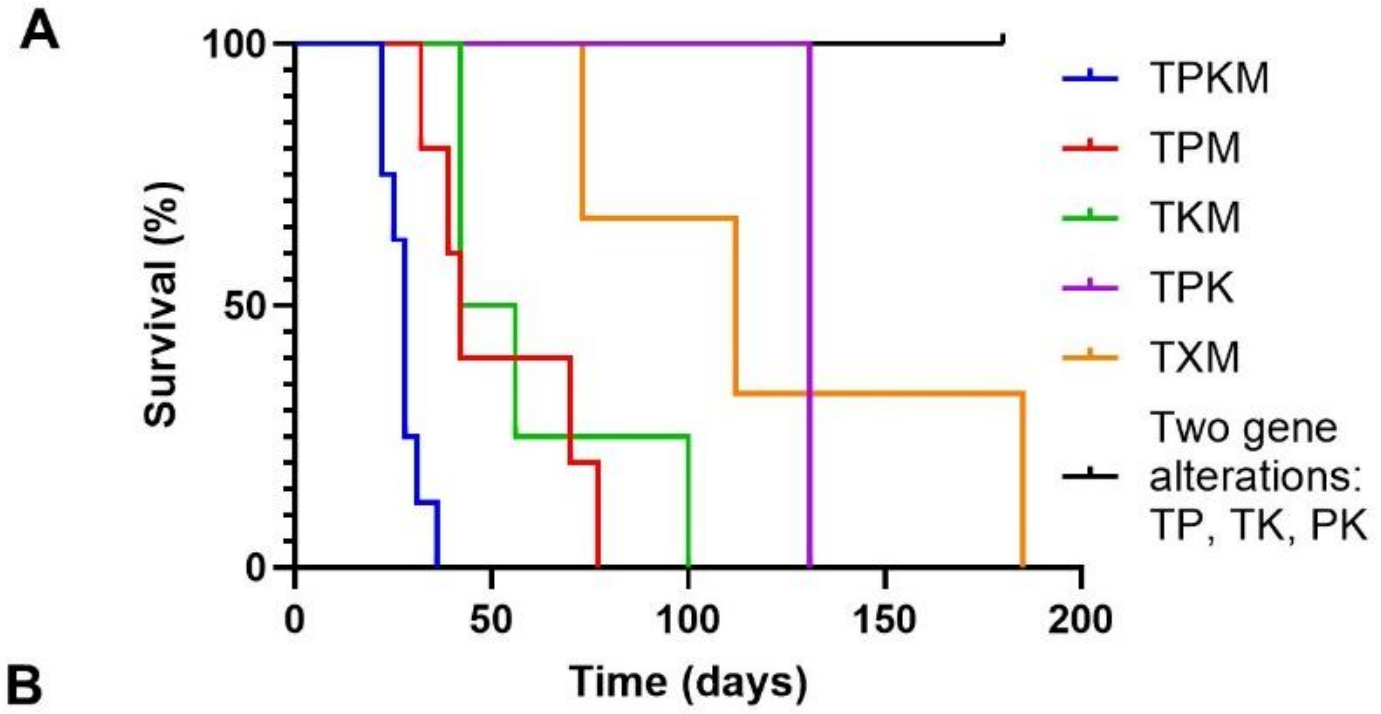


**B**

| | TPKM | | | | TPM | | | TKM | | | TPK | TXM |
|---|---|---|---|---|---|---|---|---|---|---|---|---|
| Pig # | 1 | 2 | 3 | 4 | 1 | 2 | 3 | 1 | 2 | 3 | 2 | 1 |
| Clear cell HCC | ■ | | | ■ | ■ | | | ■ | | | | |
| Trabecular HCC | ■ | ■ | ■ | ■ | ■ | | | ■ | | ■ | | ■ |
| Pseudoglandular HCC | ■ | | | ■ | | | | ■ | | ■ | | |
| Cholangiocarcinoma | ■ | ■ | ■ | ■ | ■ | ■ | ■ | ■ | ■ | ■ | | |

**C**

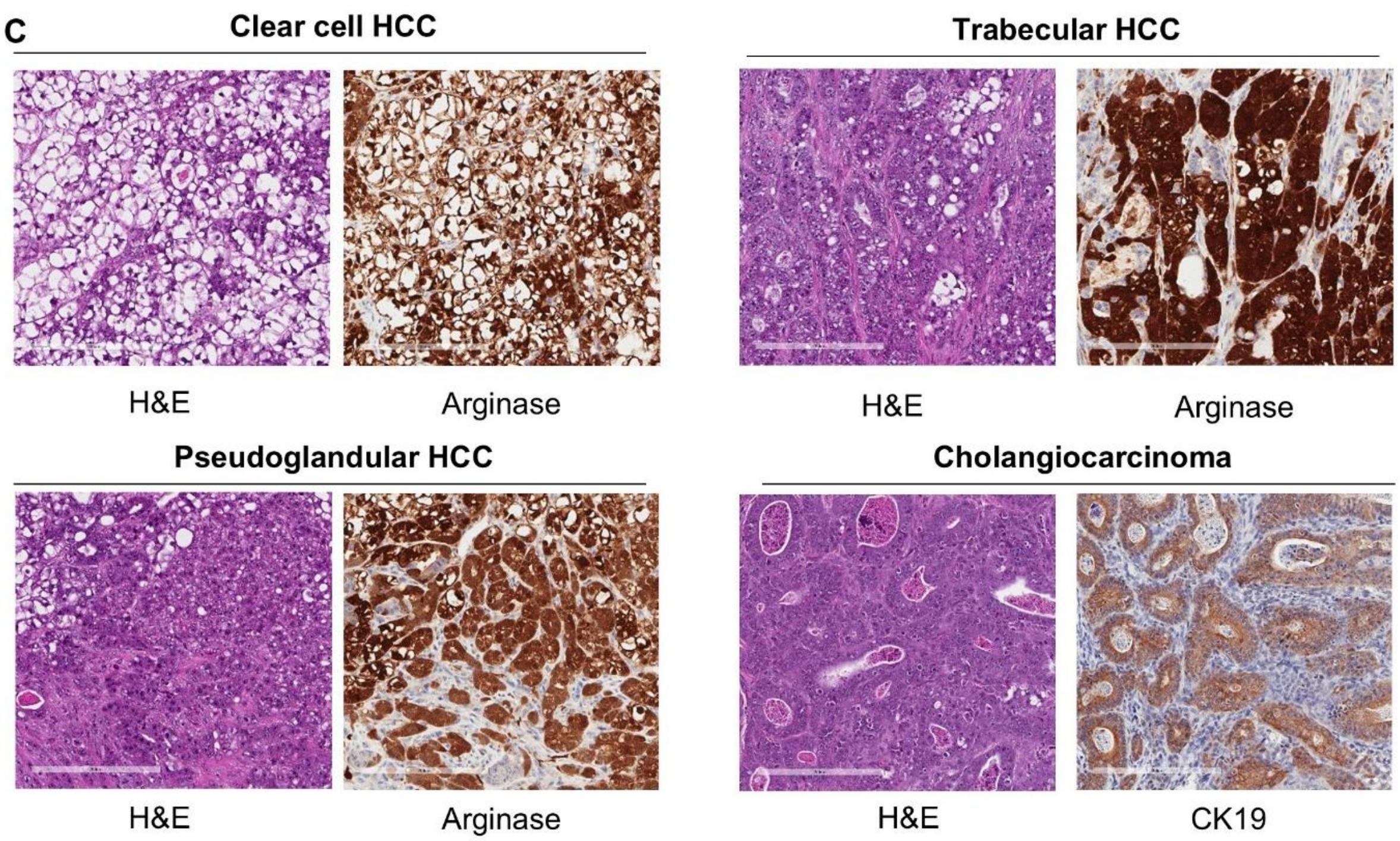


**D**

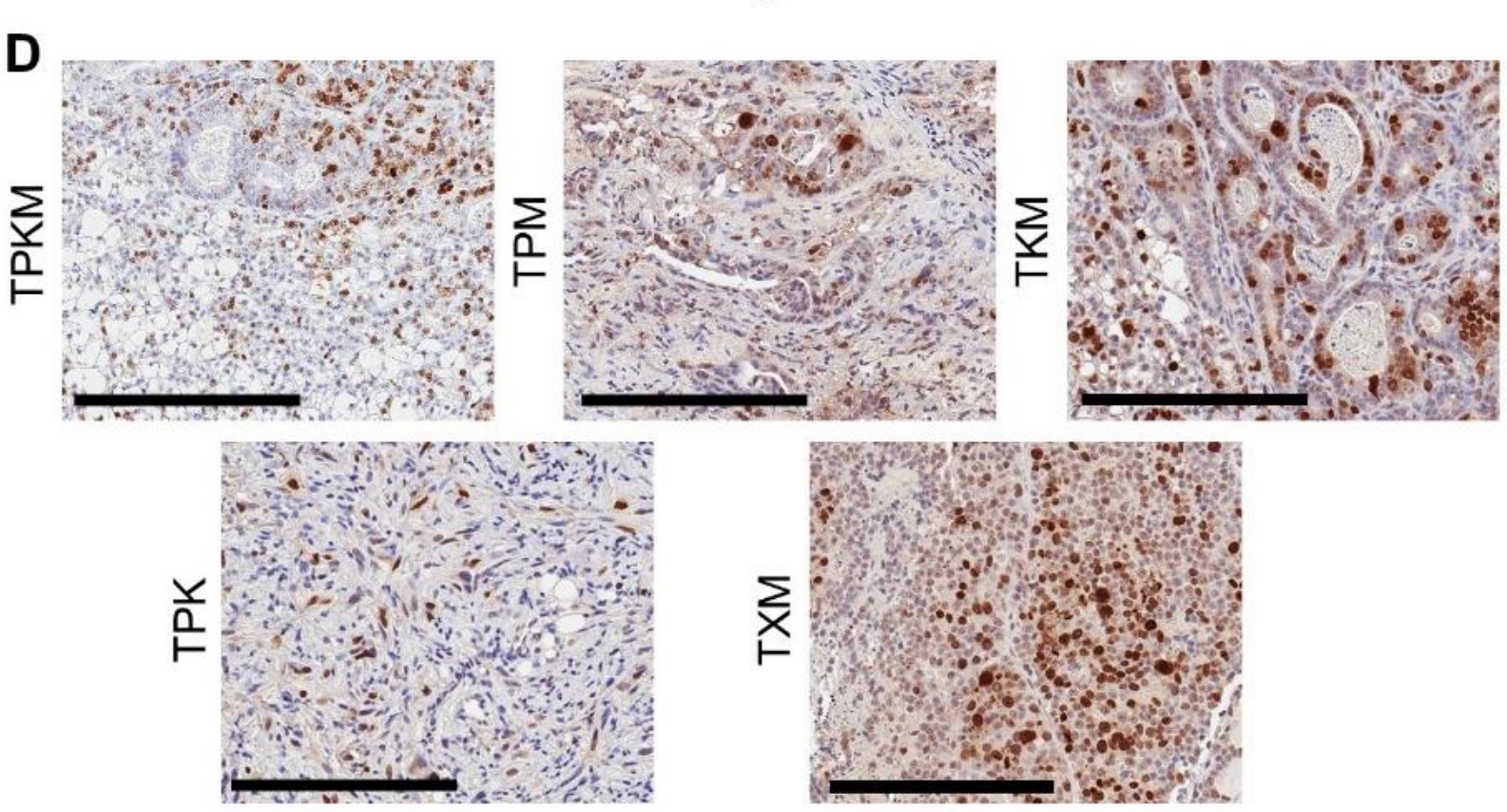


**E**

| Tumor | Ki67 proliferation index |
|---|---|
| TPKM | 25.7 |
| TPM | 26.6 |
| TKM | 32.6 |
| TPK | 7.68 |
| TXM | 17.1 |

**Fig. S4. Histopathological characterization of xenograft tumors developed by subcutaneous injection of transformed hepatocytes into SCID mice (related to Fig. 2).**

A) Kaplan–Meier survival curves of mice injected with the indicated cells. B) Heatmap indicating the presence of HCC or cholangiocarcinoma (CCA) histological features in xenograft tumors. Numbers indicate the pig of origin for each injected cell line. C) Representative TPKM tumor sections stained with H&E and Arginase or CK19, displaying features of clear cell HCC, trabecular HCC, pseudoglandular HCC, and CCA. Scale bar, 200 μm. D) Representative Ki67 staining in tumor sections. Scale bar, 300 μm. E) Ki67 proliferation index (%) quantified by digital image analysis of Ki67-stained tissue microarray cores using Halo software. Data are expressed as the percentage of Ki67-positive tumor cells.

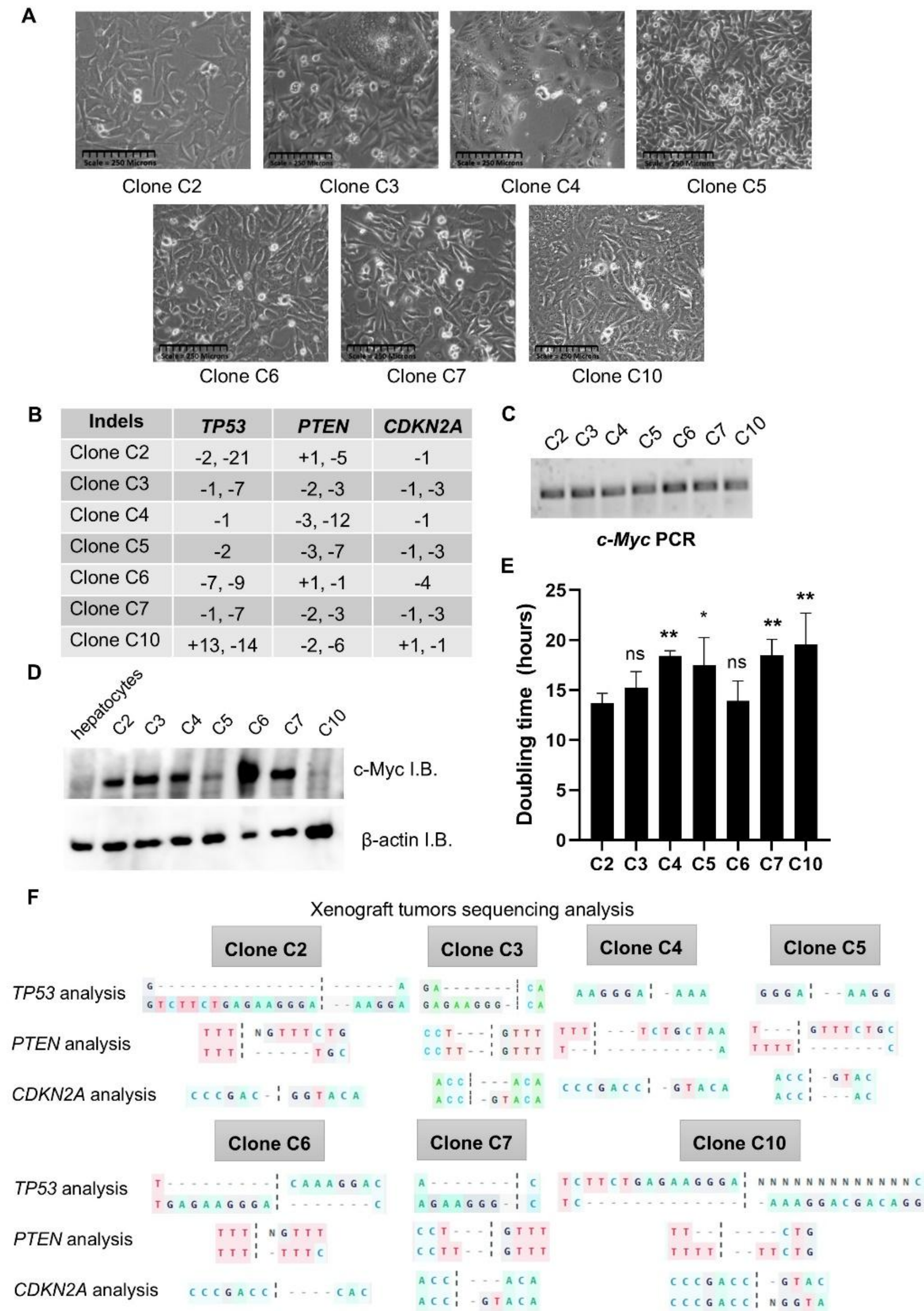


**Fig. S5. Characterization of clonal TPKM populations and corresponding xenograft tumors.**

A) Representative pictures of seven single-cell clones derived from Pig 1 TPKM cells in culture. Scale bar, 250 μm. B) Sequencing analysis confirmed the presence of CRISPR-induced indels in the targeted genes across all clones. The type of indels in each clonal population is shown. C) PCR for genomic DNA confirms *c-Myc* integration in all clones. D) Western blotting confirms c-Myc expression in the clonal populations, with variable expression levels observed. E) Doubling time (hours) of clones are presented as mean ± SD from at least three independent measurements per clone. One-way ANOVA: ns, not significant; *, $p<0.05$; **, $p<0.01$ versus C2. F) Sequencing analysis of the xenograft tumors confirmed retention of CRISPR-induced edits present in the corresponding parental cell lines. Dashed line, predicted Cas9 cleavage position; N, nucleotide insertion; dash, deleted base.

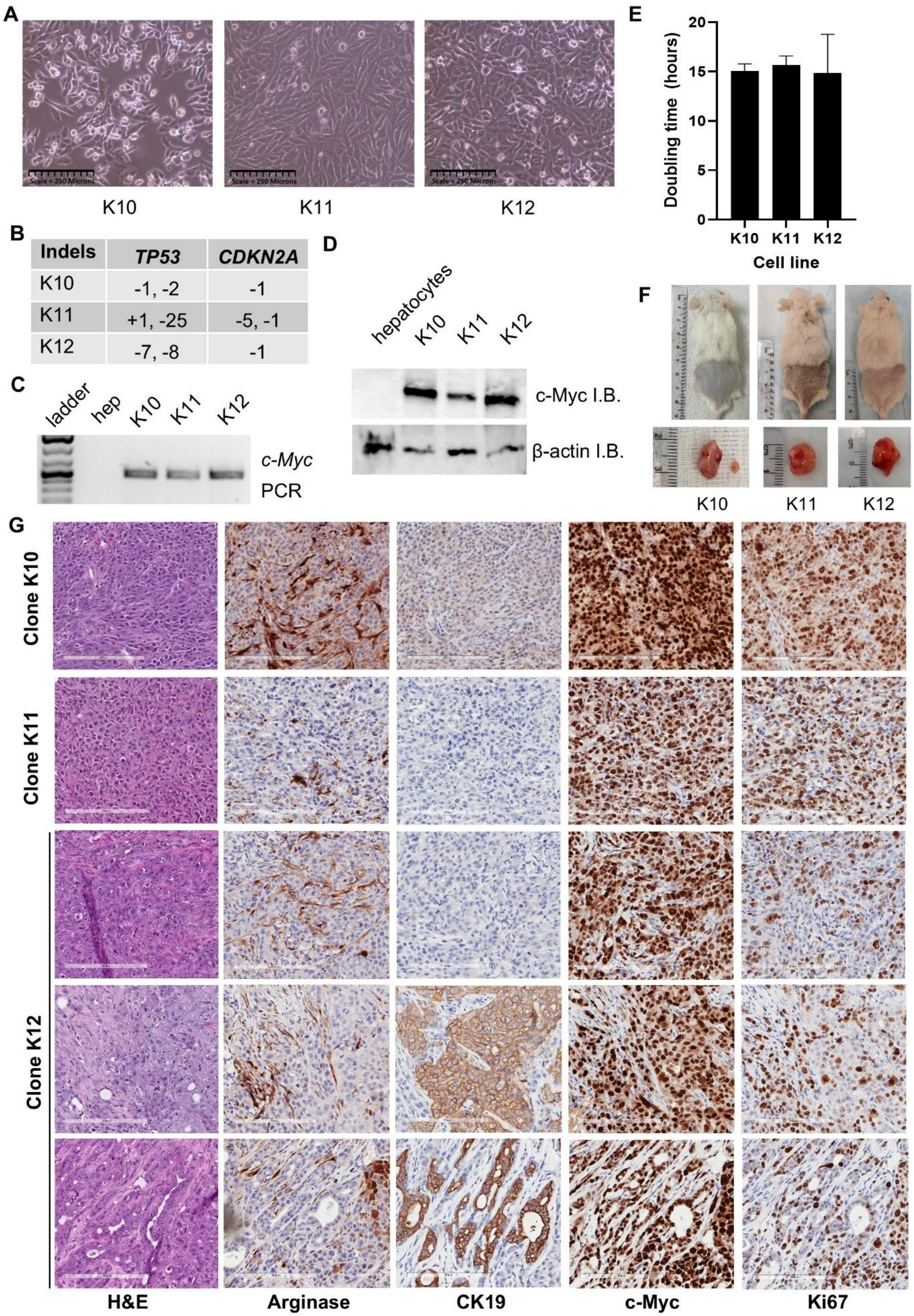


**Fig. S6. Clonal TKM cells display diverse histological features in vivo.**

A) Representative pictures of three single-cell clones isolated from Pig 1 TKM cells in culture. Scale bar, 250 µm. B) Sequencing analysis confirmed the presence of CRISPR-induced indels in *TP53* and *CDKN2A* across all clones. The type of indels in each clonal population is shown. C) PCR confirms *c-Myc* integration in all clones. D) Western blotting confirms c-Myc expression in the clonal populations, with variable expression levels observed. E) Doubling time (hours) of the cells are presented as mean ± SD from at least three independent measurements per clone. One-way ANOVA indicated no significant differences among the three clones. F) Subcutaneous injection of clonal populations into both flanks of SCID mice resulted in tumor development. Mice were euthanized when one tumor reached 1.5 cm in diameter or both tumors measured 2 cm in sum of diameters. Clone K10 resulted in the development of one large and one small tumor, while K11 and K12 clones resulted in the development of a single tumor in each mouse. G) Representative tumor sections stained with H&E, Arginase, CK19, c-Myc, and Ki67. Scale bar, 200 µm

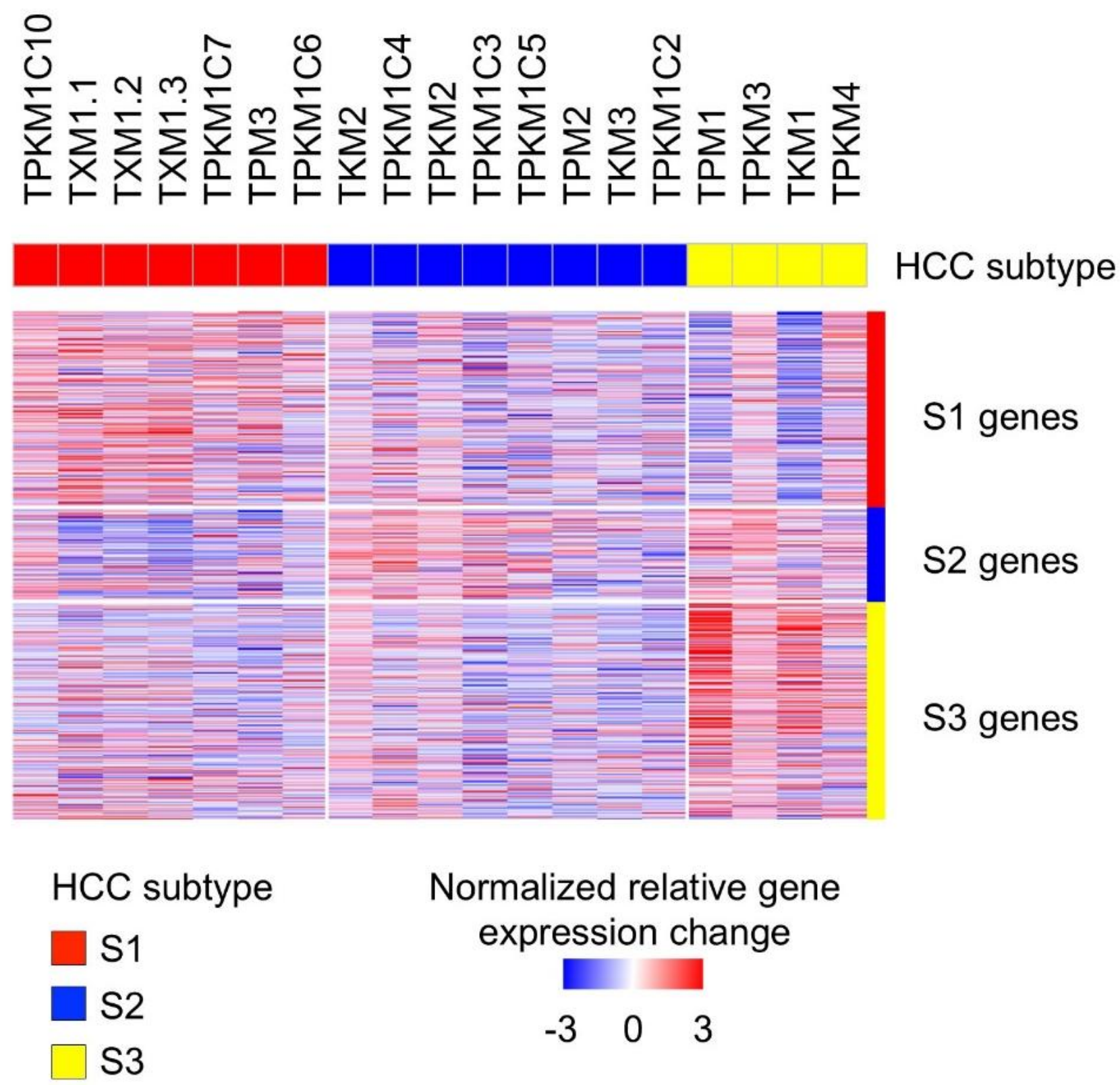


**Fig. S7. Analysis of transcriptomic meta-analysis-based human HCC subtypes in the xenograft tumors.**

Expression heatmap of the Hoshida 619-gene HCC subtype signature in TPKM, TPM, TKM, and TXM xenograft tumors. Z-score color scale is shown.

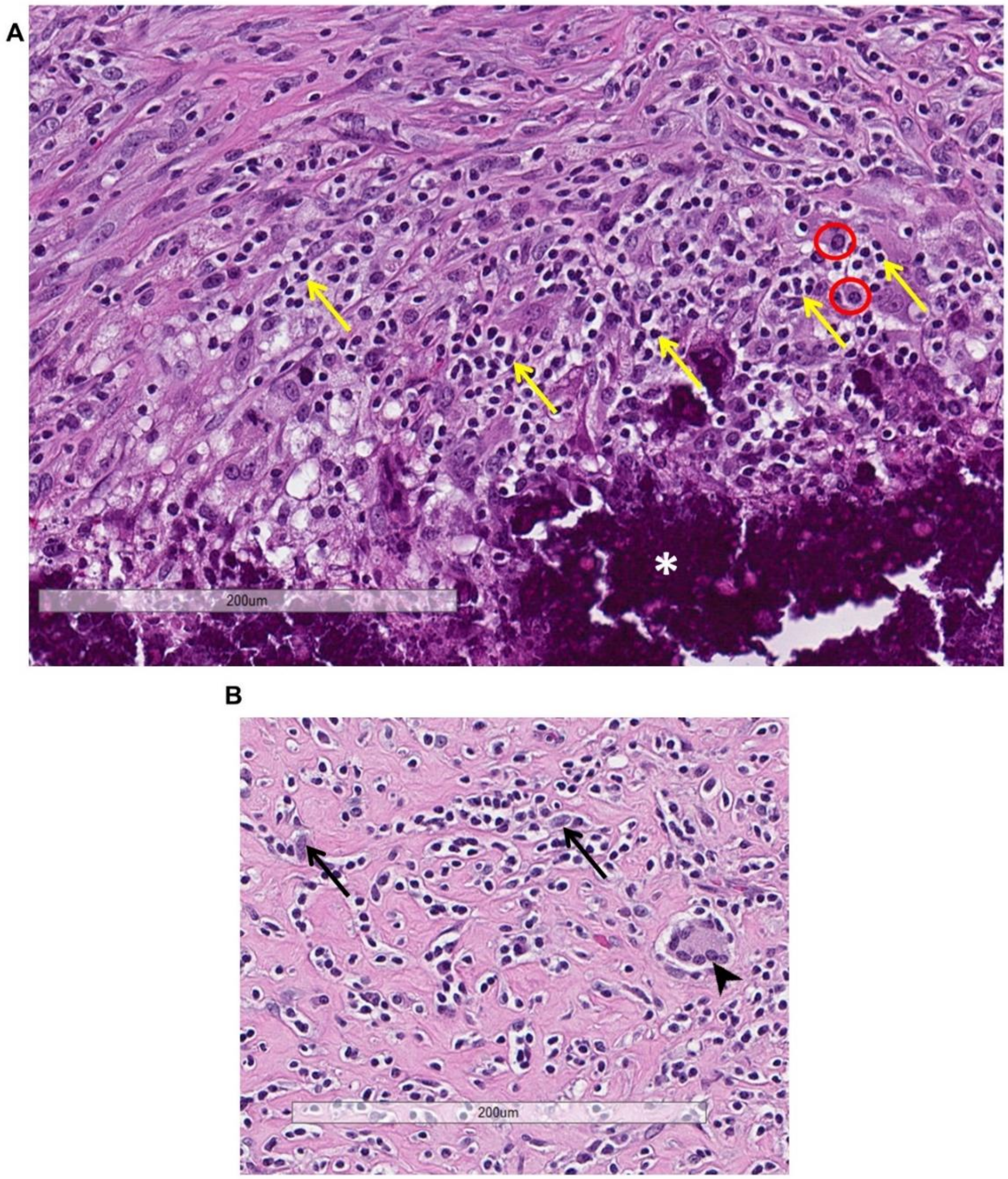


**Fig. S8. Higher-magnification H&E images demonstrating tumor immune cell infiltration in porcine liver tumors.**

A) Representative image showing lymphocytes (yellow arrows), plasma cells (red circles), and areas of calcification (white asterisk). B) Representative image showing a multinucleated giant cell (arrow head) and histiocytes (black arrows). Images are higher-magnification views of the tumor shown in **Fig. 6D**. Scale bar, 200 μm.

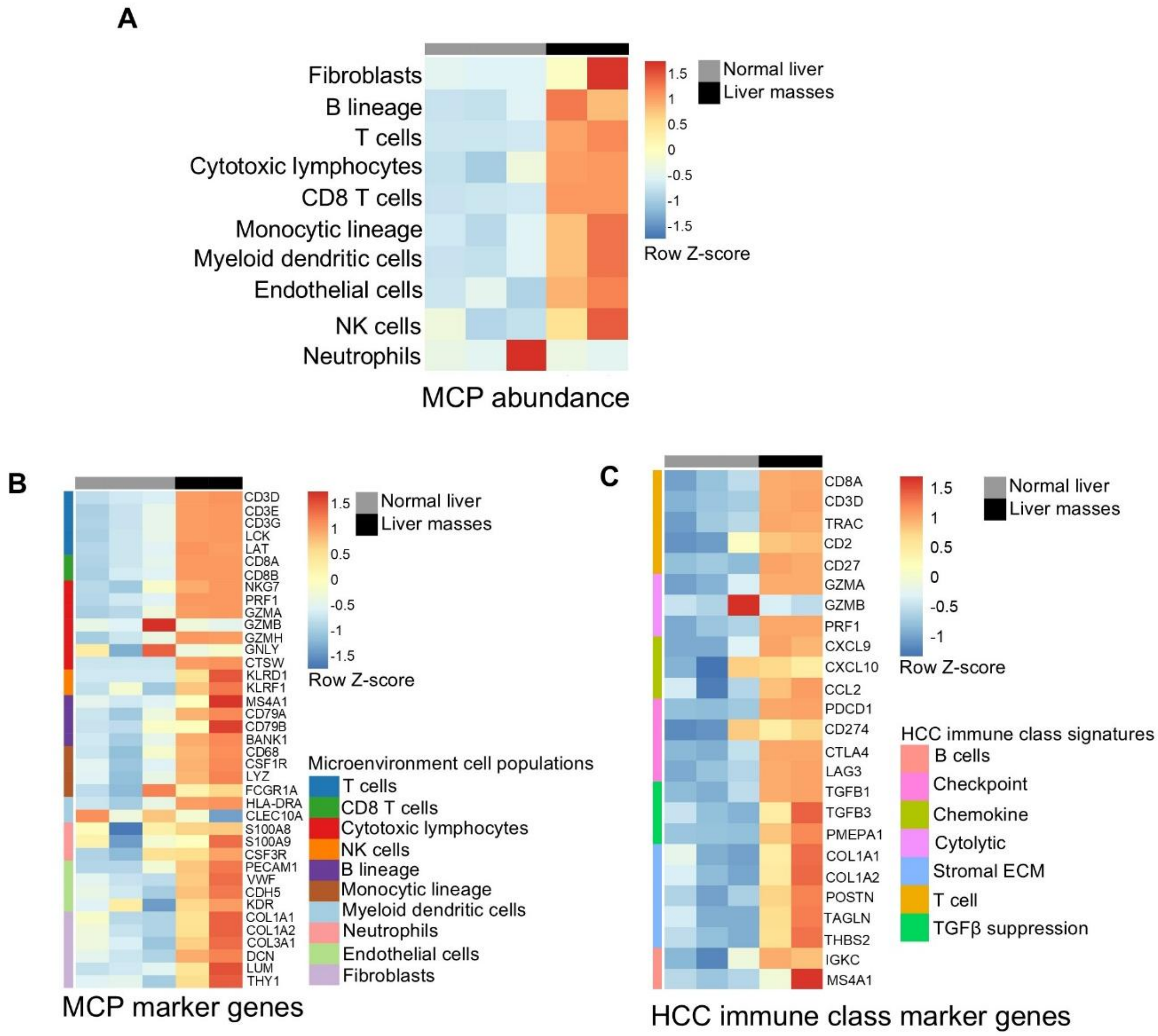


**Fig. S9. RNA sequencing-based profiling of the tumor microenvironment in porcine HCC tumors.**

A) Heatmap of row-scaled MCP-counter scores showing the relative abundance of immune and stromal cell populations across normal liver and pig tumors. B) Heatmap of MCP-counter marker gene expression displayed as row Z-scores of log2-transformed normalized counts. C) Heatmap showing relative expression of HCC immune class signature genes across normal liver and pig tumors. Row Z-scores are shown.
