# Supplementary material for: Modeling liver cancer molecular and histological features by CRISPR editing of porcine hepatocytes
Source: JHEP Rep. 2026 Sep 23;8(12):102042. doi: 10.1016/j.jhepr.2026.102042 (PMC13770662; doi:10.1016/j.jhepr.2026.102042)
Supplement: Supplementary file 4 [file mmc2.docx]

**Journal of Hepatology**

**CTAT methods**

Tables for a “Complete, Transparent, Accurate and Timely account” (CTAT) are now mandatory for all revised submissions. The aim is to enhance the reproducibility of methods.

- Only include the parts relevant to your study
- Refer to the CTAT in the main text as ‘Supplementary CTAT Table’
- Do not add subheadings
- Add as many rows as needed to include all information
- Only include one item per row

**If the CTAT form is not relevant to your study, please outline the reasons why:**

1.1 **Antibodies**

| **Name** | **Citation** | **Supplier** | **Cat no.** | **Clone no.** |
| --- | --- | --- | --- | --- |
| anti-c-Myc (for western blotting) |  | Santa Cruz Biotechnology | sc-40 | 9E10 |
| anti-actin |  | Santa Cruz Biotechnology | sc-47778 | C4 |
| anti-PTEN |  | Santa Cruz Biotechnology | Sc-7974 | A2B1 |
| Goat anti-mouse IgG-HRP secondary antibody |  | Biorad | 170-6516 |  |
| anti-Arginase-1 |  | Abcam | ab91279 |  |
| anti-CK19 |  | Developmental Studies Hybridoma Bank (DSHB) | Troma-III | Troma-III |
| anti-c-Myc (for IHC) |  | Biocare Medical | PME415AA | EP121 |
| anti-Ki67 |  | Agilent/Dako | M7240 | MIB-1 |

1.2 **Cell lines**

| **Name** | **Citation** | **Supplier** | **Cat no.** | **Passage no.** | **Authentication test method** |
| --- | --- | --- | --- | --- | --- |
| Primary porcine hepatocytes | This study | Isolated from Yucatan minipigs | N/A |  | N/A |
| Lenti-X™ 293T cells |  | Takara Bio | 632180 |  | Authenticated by supplier |

1.3 **Organisms**

| **Name** | **Citation** | **Supplier** | **Strain** | **Sex** | **Age** | **Overall n number** |
| --- | --- | --- | --- | --- | --- | --- |
| Pig |  | Premier BioSource | Yucatan minipig | male | 10-22 weeks | 7 |
| Mouse |  | Jackson Laboratory | NOD.CB17-*Prkdc^scid^*/J | male | 9-12 weeks | 37 |

1.4 **Sequence based reagents**

| **Name** | **Sequence** | **Supplier** |
| --- | --- | --- |
| *TP53* crRNA | 5’-GGTCTTCTGAGAAGGGACAA-3’ | IDT |
| *PTEN* crRNA | 5’- AGATCGTTAGCAGAAACAAA -3’ | IDT |
| *CDKN2A* crRNA | 5’- TGGCGGCGTCGTGTACCGGT -3’ | IDT |
| *AXIN1* crRNA | 5’-CCCGTCCTGATCGTCGAGCA -3’ | IDT |
| TP53-For primer | 5’- GTTGTTCCCATCCATCCGCA -3’ | IDT |
| TP53-Rev primer | 5’- GGAGAGCGAACAGAAGGTCA -3’ | IDT |
| PTEN-For primer | 5’- GAACGCCGGAGAGTTGGTCTC -3’ | IDT |
| PTEN-Rev primer | 5’- GAGACCCCGTAGACAGACTAAGA -3’ | IDT |
| CDKN2A-For primer | 5’- AGTGCAACGTTCAGGGTAACT -3’ | IDT |
| CDKN2A-Rev primer | 5’- TCTCCAGAGTTTGAGCGAGC -3’ | IDT |
| AXIN1-For primer | 5’-ACACTCACGACATGGTTCTACACACAGCTTCTGCTCTGGGAA-3’ | IDT |
| AXIN1-Rev primer | 5’-TACGGTAGCAGAGACTTGGTCTCTCCTCGTTCGAGTCGCAG-3’ | IDT |
| c-myc-For primer | 5’-CTCTCCGTCCTCGGATTCTC-3’ | IDT |
| c-myc-Rev primer | 5’-GCTCCGTTTTAGCTCGTTCC-3’ | IDT |
|  |  |  |
|  |  |  |

1.5 **Biological samples**

| **Description** | **Source** | **Identifier** |
| --- | --- | --- |
| Porcine liver tissue | This study |  |
| Xenograft/porcine tumors | This study |  |
|  |  |  |
|  |  |  |

1.6 **Deposited data**

| **Name of repository** | **Identifier** | **Link** |
| --- | --- | --- |
| Gene Expression Omnibus (GEO) | GSE311743 | https://www.omicsdi.org/dataset/geo/GSE311743 |

1.7 **Software**

| **Software name** | **Manufacturer** | **Version** |
| --- | --- | --- |
| FASTQC | Babraham Bioinformatics | v0.12.0 |
| Trimmomatic | Usadel laboratory/Open source | v0.36 |
| STAR | Dobin laboratory/Open source | v2.7.11b |
| RSEM | Li & Dewey/Open source | v1.3.3 |
| DEBrowser | Bioconductor/Open source | v1.34.0 |
| Aperio ImageScope | Leica | 12.4.0.5043 |
| Ingenuity Pathway Analysis (IPA) | QIAGEN Digital Insights |  |
| ICE (Inference of CRISPR Edits) | Synthego | Web tool |
| Halo | Indica Labs | v4.0.5107.488 |
| GraphPad Prism | GraphPad Software | V11 |

1.8 **Other (e.g. drugs, proteins, vectors etc.)**

| pCDH-Myc-Puro plasmid | Addgene | 46970 |
| --- | --- | --- |
| psPAX2 plasmid | Addgene | 12260 |
| pMD2.G plasmid | Addgene | 12259 |
|  |  |  |
|  |  |  |

1.9 **Please provide the details of the corresponding methods author for the manuscript:**

Lobna Elkhadragy, PhD

Department of Radiology, University of Illinois Chicago

2201 W Campbell Park Drive, Suite 210

Chicago, IL 60612, USA

e-mail: lobna@uic.edu

**2.0	Please confirm for randomised controlled trials all versions of the clinical protocol are included in the submission. These will be published online as supplementary information.**

**N/A**
